# The Epstein-Barr Virus Immunoevasins BCRF1 and BPLF1 Are Expressed by a Mechanism Independent of the Canonical Late Pre-initiation Complex

**DOI:** 10.1371/journal.ppat.1006008

**Published:** 2016-11-17

**Authors:** Jessica McKenzie, Francesc Lopez-Giraldez, Henri-Jacques Delecluse, Ann Walsh, Ayman El-Guindy

**Affiliations:** 1 Department of Pediatrics Yale University School of Medicine, New Haven, Connecticut, United States of America; 2 Yale Center for Genome Analysis (YCGA), Yale University, West Haven, Connecticut, United States of America; 3 Department of Tumor Virology, German Cancer Research Center, Im Neuenheimer Feld, Heidelberg, Germany; Baylor College of Medicine, UNITED STATES

## Abstract

Subversion of host immune surveillance is a crucial step in viral pathogenesis. Epstein-Barr virus (EBV) encodes two immune evasion gene products, BCRF1 (viral IL-10) and BPLF1 (deubiquitinase/deneddylase); both proteins suppress antiviral immune responses during primary infection. The BCRF1 and BPLF1 genes are expressed during the late phase of the lytic cycle, an essential but poorly understood phase of viral gene expression. Several late gene regulators recently identified in beta and gamma herpesviruses form a viral pre-initiation complex for transcription. Whether each of these late gene regulators is necessary for transcription of all late genes is not known. Here, studying viral gene expression in the absence and presence of siRNAs to individual components of the viral pre-initiation complex, we identified two distinct groups of late genes. One group includes late genes encoding the two immunoevasins, BCRF1 and BPLF1, and is transcribed independently of the viral pre-initiation complex. The second group primarily encodes viral structural proteins and is dependent on the viral pre-initiation complex. The protein kinase BGLF4 is the only known late gene regulator necessary for expression of both groups of late genes. ChIP-seq analysis showed that the transcription activator Rta associates with the promoters of eight late genes including genes encoding the viral immunoevasins. Our results demonstrate that late genes encoding immunomodulatory proteins are transcribed by a mechanism distinct from late genes encoding viral structural proteins. Understanding the mechanisms that specifically regulate expression of the late immunomodulatory proteins could aid the development of antiviral drugs that impair immune evasion by the oncogenic EB virus.

## Author Summary

Late proteins are expressed during the productive cycle of Epstein-Barr virus (EBV) after the onset of viral DNA replication. Many late proteins serve structural functions; they form the capsid shell around the viral genome or mediate attachment and fusion of the virus to the host cell. EBV also encodes two late proteins that suppress the immune system during primary infection. The current model suggests that transcription of all late genes is regulated by a common mechanism involving seven late gene regulators. Here, we demonstrate that late genes encoding two viral immune suppressants are transcribed by a mechanism different from that regulating late genes encoding structural proteins. Abolishing expression of the late immunomodulators without disrupting expression of the antigenic viral structural proteins could serve as an approach to block EBV *de novo* infection and its associated malignancies.

## Introduction

Late genes represent more than one third of the herpesvirus genome. The functions of many of these genes are indispensable for the life cycle of the virus. Late genes encode structural proteins that form the viral capsid, and glycoproteins that mediate virus attachment, fusion and entry during primary infection. Other late proteins also mediate essential events during virion assembly and maturation such as viral DNA cleavage and packaging into pre-formed capsids, capsid envelopment, and egress of infectious particles. Furthermore, late proteins play an integral role in suppressing the immunogenicity of infected cells.

Here we investigate the expression of late genes in Epstein-Barr virus (EBV), an oncogenic gamma herpesvirus associated with several forms of cancer including Burkitt lymphoma [1], nasopharyngeal carcinoma [2, 3], Hodgkin lymphoma [4], gastric carcinoma [5, 6], post-transplant lymphoproliferative disease [7, 8], and AIDS-associated lymphoma [9]. Currently, there are no drugs or vaccines that can interfere with EBV primary infection. Studying the mechanisms that regulate the various phases of the virus life cycle is crucial to generate new means to control EBV infection and its associated diseases.

While late products play an essential role in the life cycle of EBV, key players regulating their expression have only been recently identified. We described two EBV-encoded proteins that regulate synthesis of late mRNAs [10]. These two late gene regulators are BGLF3, an early protein that has no cellular homologs or identifiable domains, and BGLF4, a serine/threonine protein kinase conserved in all herpesviruses. Knockdown of BGLF3 or BGLF4 selectively abolished expression of late genes independent of any effect on expression of early genes or viral DNA replication [10]. The mechanism by which BGLF4 regulates expression of late genes is not clear; however, the kinase activity of BGLF4 is indispensable for accumulation of late products. BGLF3 is part of a viral pre-initiation complex (vPIC) dedicated for transcription of late genes [11]. The complex contains at least five additional early lytic proteins. These proteins are: BcRF1, BDLF3.5, BDLF4, BFRF2, and BVLF1. Knockout of BcRF1, BDLF4, BVLF1, BDLF3.5 and BFRF2 disrupts expression of late genes [11–14]. However, the exact function of several of these late gene regulators is still unknown.

The current model suggests that late gene regulators assemble together to form a viral pre-initiation complex (vPIC) that mediates recruitment of the RNA polymerase II complex (RNAPII) to late promoters. In support of this model, disrupting expression of the murine gamma herpesvirus BGLF3 or BDLF3.5 orthologs reduced association of the large catalytic subunit (RPB1) of RNAPII to late promoters [15]. Selective recognition of late promoters is mediated by the late gene regulator BcRF1, a viral protein predicted to have a saddle-like protein-fold that is characteristic of the cellular TATA-box binding protein (TBP) [13, 16]. The binding specificity of BcRF1 (vTBP) is different than cellular TBP. BcRF1 preferentially binds to a TATT element (with a T rather than A at the fourth position) that is present in most late promoters [13, 17]. BcRF1 directly interacts with subunits of RNAPII and recruits the polymerase complex to late promoters [11]. Similar observations were reported for ORF24, the ortholog of vTBP in Kaposi’s sarcoma-associated herpesvirus [18]. Mutations in ORF24 that disrupt its interaction with RPB1 or the TATT sequence present in late promoters abolished synthesis of late transcripts [19]. HCMV UL79, the ortholog of EBV BVLF1, functions as an elongation factor of RNAPII during the late phase of viral gene expression [20]. Sub-nuclear localization studies revealed that late gene regulators assemble in replication compartments and that true late genes are transcribed from newly replicated viral DNA [14, 21, 22]. Formation of replication compartments might increase the local concentration of these late gene regulators and promote their interaction with replication proteins to stimulate synthesis of late transcripts.

Despite recent advances in identifying virally encoded late gene regulators, many questions remain. One specific question that we address here is whether the same set of late gene regulators activates expression of all late genes. In this study we began by knocking down expression of BGLF3 and examined the EBV transcriptome during the late phase of lytic infection. We found that transcript levels of several late genes were not altered by the absence of BGLF3. We focused on four of these BGLF3-independent genes that are expressed with true late kinetics. We demonstrated that expression of this subclass of late genes is regulated by the BGLF4 protein kinase but not by other known late gene regulators. Interestingly, two of the BGLF3-independent late genes, BCRF1 and BPLF1, function as immunomodulators. BCRF1, a homolog of human IL10 (vIL10), and BPLF1, a deneddylase/deubiquitinase (vDUB) protein, suppress immune recognition during the productive lytic cycle and viral *de novo* infection [23–27]. Our findings show that the mechanism regulating expression of EBV antigenic late structural proteins differs from that of late immunosuppressants.

## Results

### Gene expression profiling reveals a new subclass of late genes transcribed in the absence of BGLF3

**Fig 1:**
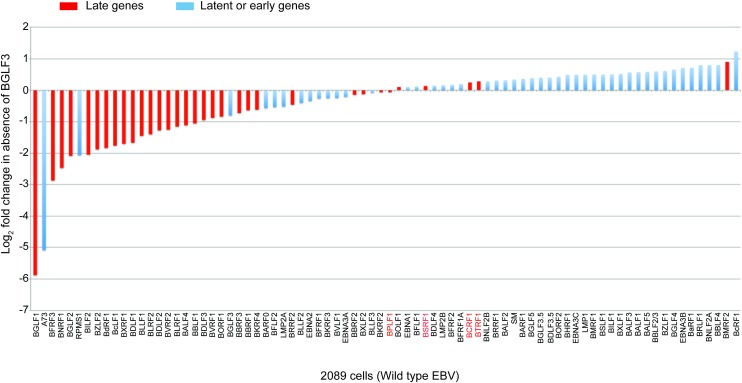
Knockdown of BGLF3 spares the expression of a subgroup of late genes. Comparison of the EBV transcriptome in the absence and presence of siRNA to BGLF3 (siBGLF3). Deep sequence analysis was performed on mRNA purified from ZEBRA-transfected 2089 cells at a late time point (48h) during lytic infection. Red bars represent fold-change in expression of late genes; blue bars represent latent and early genes. Fold-change in expression was calculated as the ratio of the number of normalized reads per transcript in the presence of siBGLF3 to the number of reads in the absence of siBGLF3. BPLF1, BSRF1, BCRF1 and BTRF1 represent lytic EBV genes previously categorized as late but were expressed in the absence of the late gene regulator, BGLF3. This subclass of genes is referred to as BGLF3-independent late genes.

Recently we identified BGLF3 as a lytic product essential for transcription of late genes from the endogenous viral genome [10]. Silencing expression of BGLF3 selectively reduced the level of three late transcripts, BFRF3 (minor viral capsid protein), BcLF1 (major viral capsid protein) and BdRF1 (scaffold protein), but had no effect on the level of an early transcript, BMRF1 (the polymerase processivity factor), or the capacity of the virus to amplify its genome [10]. To determine which lytic genes require the function of BGLF3 for their expression, we studied the EBV transcriptome in the absence and presence of siRNA to BGLF3 (siBGLF3). We transfected 2089 cells, 293 cells harboring wild type EBV bacmid, with an expression vector encoding ZEBRA, the lytic cycle activator, either alone or together with 50 nM siBGLF3. A Western blot analysis was performed 48h after transfection to confirm induction of the lytic cycle in cells transfected by ZEBRA. Co-transfection of siBGLF3 markedly reduced the level of late FR3 protein, in accordance with our previous data [10]. Total RNA was purified and subjected to RNA-seq analysis. The relative abundance of each transcript was estimated as the number of reads mapping to a particular gene normalized to the total number of viral reads [28, 29]. Fig 1 compares the abundance of each transcript in the absence and presence of siBGLF3. The change in expression is represented as a log base 2-fold. The kinetic classes of EBV genes were assigned based on viral gene classification reported by Yuan et al [30]. Viral transcripts with a negative fold-change represent EBV genes whose expression is dependent on the presence of BGLF3, and vice versa. We found that silencing of BGLF3 reduced expression of 23 late genes. This result supports our previous conclusion that BGLF3 is a late gene regulator [10]. However, the level of several late transcripts, e.g. BTRF1 (tegument protein), BPLF1 (vDUB), BCRF1 (vIL10), and BSRF1 (tegument protein, homolog of HHV1 UL51, an egress protein), was not affected by the lack of BGLF3 (Fig 1 and S1 Fig). We refer to these genes as the BGLF3-independent late genes. This finding suggests that some late genes might be expressed in a manner independent of BGLF3. Thus, expression of late genes could be regulated by more than one mechanism.

### A subset of EBV late genes is expressed in the absence of BGLF3

**Fig 2:**
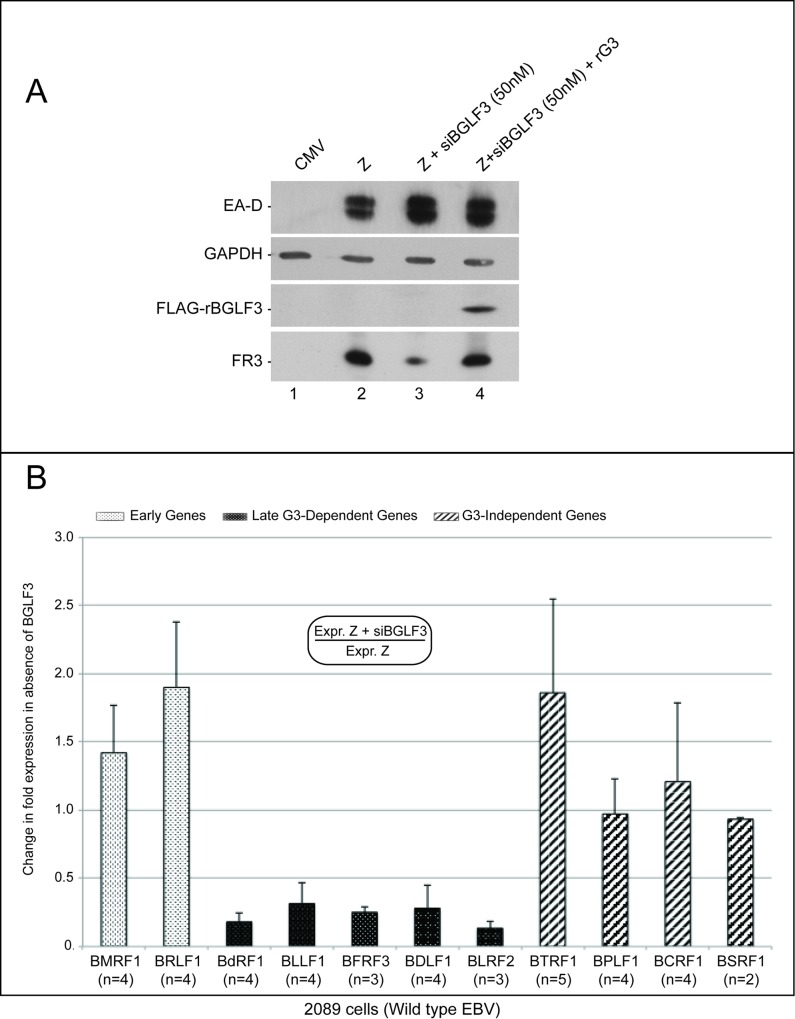
A subclass of late genes is expressed in the absence of BGLF3. A) Western blot analysis of 2089 cell lysates demonstrating the effect of siBGLF3 on expression of EBV early (BMRF1) and late (FR3) proteins. The effect of siBGLF3 on expression of the late FR3 protein was suppressed by expressing a form of BGLF3 resistant to the siRNA (rBGLF3) (lane 4). B) The effect of siBGLF3 on expression of early and late EBV genes was analyzed by RT-qPCR. 2089 cells were transfected with ZEBRA or ZEBRA plus siBGLF3 and harvested at 48h after transfection. Change in expression was calculated by dividing the level of each transcript in the absence of BGLF3 by the level of the same transcript in the presence of BGLF3. Dotted white bars represent early genes, dotted black bars represent late genes, and diagonally stripped bars denote BGLF3-independent genes. The number of biological replicates is indicated for each transcript (n). FR3, BFRF3; Z, ZEBRA, and siBGLF3, siRNA to BGLF3.

Our RNA-seq data suggests that BGLF3 is dispensable for expression of several lytic genes that were previously categorized as late (Fig 1) [30]. To verify this result we used RT-qPCR to compare expression of four BGLF3-independent genes (BTRF1, BPLF1, BCRF1, and BSRF1) with two early genes, BMRF1 and BRLF1 (transcription activator), and five BGLF3-dependent late genes, BdRF1 (scaffold protein), BLLF1 (glycoprotein gp350), BFRF3 (minor viral capsid protein), BDLF1 (triplex capsid protein), and BLRF2 (tegument protein), with and without siBGLF3. Selection of the four BGLF3-independent late genes was based on our capacity to establish their true late kinetics using RT-qPCR. The comparison was performed in 2089 cells transfected with empty vector (CMV), ZEBRA (Z), or ZEBRA plus siBGLF3 (50 nM). Expression of ZEBRA in 2089 cells triggered the lytic cycle leading to accumulation of the early BMRF1 and the late FR3 proteins (Fig 2A lane 2). Silencing expression of BGLF3 reduced the level of FR3 but had no effect on the protein level of BMRF1 (Fig 2A lane 3). To demonstrate that the effect of siBGLF3 on late gene expression is specific to silencing BGLF3 rather than an off-target activity, we inserted silent mutations to generate a form of BGLF3 that is resistant to the siRNA (rBGLF3). Co-transfection of rBGLF3 in lytic 2089 cells suppressed the effect of siBGLF3 on synthesis of late products and restored expression of the late FR3 protein (Fig 2A lane 4 and [10]).

Fig 2B compares expression of eleven lytic transcripts in the absence and presence of BGLF3. Change in expression was calculated as the amount of mRNA detected in cells transfected with ZEBRA or ZEBRA plus siBGLF3 (50 nM) relative to empty vector. The relative concentration of each transcript was corrected according to the level of GAPDH mRNA measured in the same sample. Knockdown of BGLF3 expression did not reduce the level of the two early transcripts, BRLF1 and BMRF1 (Fig 2B); instead a slight increase was detected. In agreement with the RNA-seq data presented in Fig 1, knockdown of BGLF3 significantly reduced the level of the five BGLF3-dependent late genes but did not significantly decrease expression of the four BGLF3-independent late genes (Fig 2B and S1 Table). This result supports the model for the presence of at least two subclasses of late genes that differ in their mechanisms of expression based on the requirement of BGLF3.

### Four BGLF3-independent genes are expressed with true late kinetics

**Fig 3:**
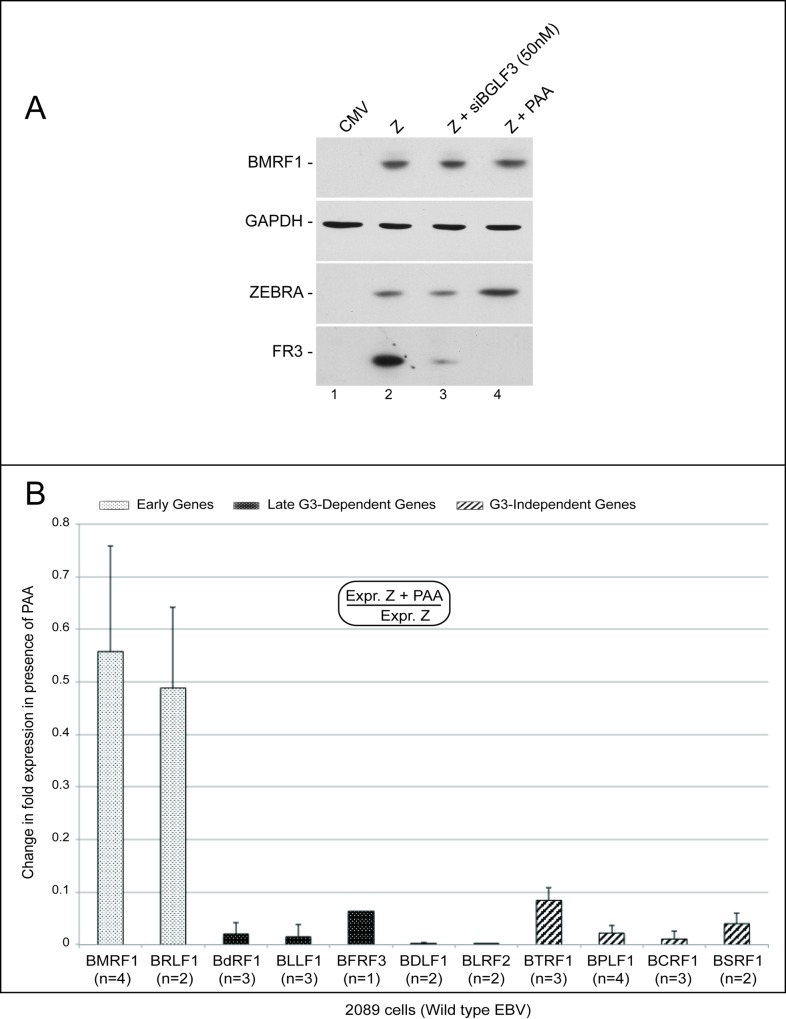
Phosphonoacetic acid inhibits expression of four BGLF3-independent genes. A) Western blot analysis of 2089 cell lysates assessing the effect of siBGLF3 and phosphonoacetic acid (PAA), an inhibitor of viral DNA replication, on expression of viral genes. B) The effect of phosphonoacetic acid (PAA) on expression of EBV early (dotted white bars), late (dotted black bars) and BGLF3-independent (diagonally stripped bars) genes in lytic 2089 cells. RT-qPCR was used to assess the level of each transcript. BTRF1, BPLF1, BCRF1 and BSRF1 represent a new subclass of late genes expressed in a manner independent of BGLF3. The value of (n) indicates the number of biological replicates. FR3, BFRF3; Z, ZEBRA, and siBGLF3, siRNA to BGLF3.

BGLF3 reportedly functions in a viral pre-initiation complex dedicated to transcription of late genes [11]. Thus, it was imperative to assess the kinetics of expression of the BGLF3-independent genes. Typically, EBV late genes are synthesized only after the onset of viral DNA replication. To examine temporal expression of the BGLF3-independent genes, we transfected 2089 cells with ZEBRA in the absence or presence of an inhibitor of viral DNA replication, phosphonoacetic acid (PAA) [31]. Addition of PAA to 2089 cells transfected with ZEBRA blocked expression of the true late FR3 protein (Fig 3A, compare lanes 2 and 4). However, PAA treatment did not reduce the level of ZEBRA or the early BMRF1 protein.

Using RT-qPCR, we compared expression of eleven lytic genes: four BGLF3-independent, two early and five BGLF3-dependent late genes (Fig 3B). Fold expression was calculated as the ratio of a particular transcript in lytic 2089 cells transfected with ZEBRA versus non-lytic cells transfected with empty vector. The level of a particular mRNA in the absence or presence of PAA was corrected to the level of GAPDH in the same sample. PAA treatment reduced the level of all eleven mRNAs regardless of their kinetic class. However, the effect of PAA on expression of BGLF3-dependent late genes was drastically more reduced than its effect on early genes. Addition of PAA to lytic cells decreased early gene expression to an average of 52% but BGLF3-dependent late genes to an average of 2% relative to lytic cells without PAA. Expression of the four BGLF3-independent late genes (BTRF1, BPLF1, BCRF1 and BSRF1) was markedly reduced to an average of 4% relative to ZEBRA-transfected 2089 cells not treated with PAA. These results with PAA strongly indicate that at least four of the BGLF3-independent genes were expressed with true late kinetics.

To confirm the late kinetics of BTRF1, BPLF1, BCRF1 and BSRF1, we studied their expression in delta BMRF1 cells (293 cells carrying EBV-bacmid with deletion in the BMRF1 gene). BMRF1 is a component of the viral DNA polymerase holo-enzyme [32] and is indispensable for viral genome amplification (S2A Fig and [33]). Expression of ZEBRA in delta BMRF1 cells induced synthesis of the two early transcripts BRLF1 and BBLF2/3 (S2B and S2C Fig), but failed to induce synthesis of late BLLF1 gene (S2D Fig) and the four BGLF3-independent genes (S2E–S2H Fig). Co-expression of ZEBRA and BMRF1 restored viral DNA replication, expression of the late FR3 viral capsid protein (S2A Fig) and synthesis of late transcripts including the four studied BGLF3-independent genes (S2D–S2H Fig). These results provide strong evidence that BTRF1, BPLF1, BCRF1 and BSRF1 are true late genes expressed in the absence of the BGLF3 late gene regulator.

### Silencing expression of the EBV late gene regulators maintains viral DNA replication but selectively abolishes synthesis of late products

**Fig 4:**
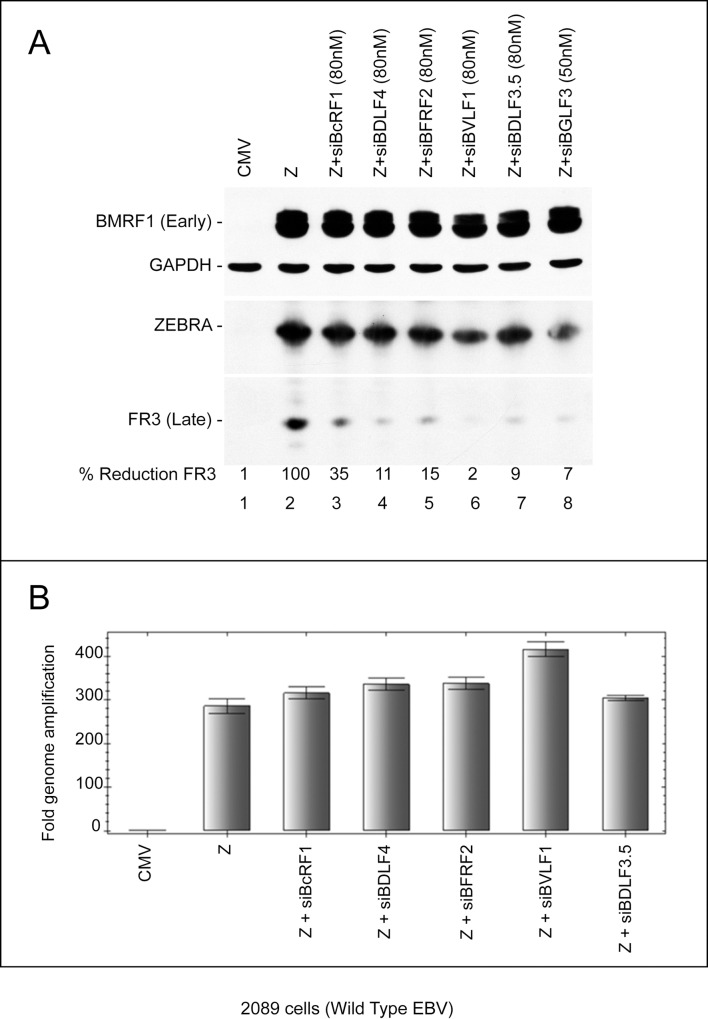
Knockdown of individual late gene regulators disrupts expression of the late FR3 protein without affecting viral DNA replication. A) Western blot analysis of cell lysates prepared from 2089 cells transfected with empty vector, ZEBRA or ZEBRA together with siRNAs towards BcRF1, BDLF4, BFRF2, BVLF1, BDLF3.5, and BGLF3. The membrane was probed with antibodies against BMRF1 (early protein), BFRF3 (late protein) and GAPDH as a loading control. B) Quantitative measurement of the amount of viral genome amplification in each condition using real time PCR. The amount of DNA per sample was corrected for the level of 18S DNA. FR3, BFRF3, and Z, ZEBRA.

In addition to BGLF3, EBV encodes five other late gene regulators that were previously shown to be essential for activation of late promoters in reporter assays [11]. These proteins are: BcRF1, BDLF4, BFRF2, BVLF1 and BDLF3.5. All six late gene regulators are thought to assemble on late promoters to form a distinct viral pre-initiation complex (vPIC) for transcription of late genes [11]. As illustrated in Fig 4, we used siRNAs specific to each of the five late gene regulators to study their roles in activation of late genes from the endogenous viral genome. The capacity of these siRNAs to disrupt expression of late genes was studied in 2089 cells transfected with ZEBRA (Z), or ZEBRA plus siRNA. We selected siRNAs that had no effect on expression of the early BMRF1 protein but markedly reduced expression of the late FR3 protein (Figs 4A and S3). Using RT-qPCR, we examined the capacity of the selected siRNAs to knockdown expression of their corresponding mRNA (S4 Fig). The specificity of the selected siRNAs was further assessed by generating siRNA-resistant forms of BcRF1 (rBcRF1), BDLF4 (rBDLF4), BFRF2 (rBFRF2), and BVLF1 (rBVLF1). Expression of rBcRF1, rBDLF4, rBFRF2, and rBVLF1 suppressed the effect of the corresponding siRNA on synthesis of the late FR3 protein (S5 Fig). The specificity of siBGLF3 and siBGLF4 was previously established using a similar approach (Fig 2A and [10]). These results demonstrate that the effect of each of the selected siRNAs on the late phenotype was specific to silencing expression of its corresponding late gene regulator. Altogether, our data suggest that BcRF1, BDLF4, BFRF2, BVLF1, and BDLF3.5 are individually essential for expression of late genes from the endogenous virus genome; none of these regulators play a redundant role in synthesis of late products.

To determine whether any of the late gene regulators affect the process of viral genome amplification, we purified DNA from 2089 cells transfected with ZEBRA alone or together with 80 nM of each siRNA. Using qPCR, we found that transfection of ZEBRA increased the level of intracellular viral DNA by 286-fold relative to cells transfected with empty vector. None of the siRNAs to late gene regulators compromised the capacity of the virus to amplify its genome (Fig 4B).

### Knockdown of individual late gene regulators abolishes production of new virus particles

**Fig 5:**
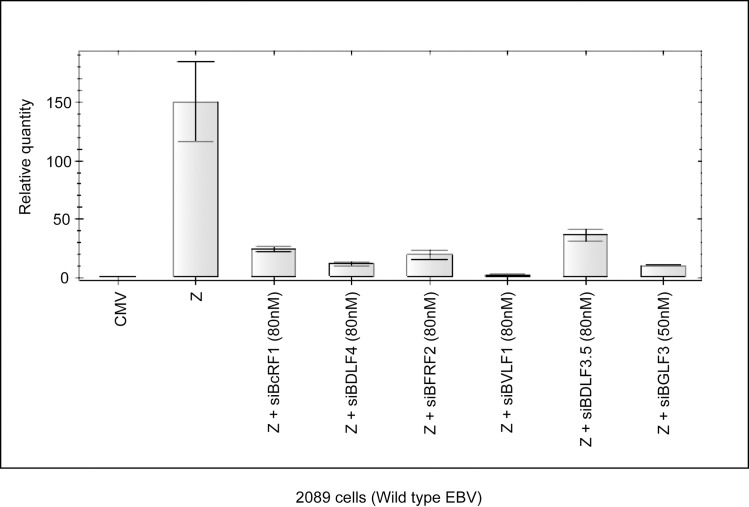
Silencing expression of individual late gene regulators reduced virion production. The level of extracellular DNA enclosed in virus particles was assessed by quantitative PCR. Cell culture media from samples used to generate Fig 4 were digested with DNase-1 to remove free extracellular DNA. Virion DNA was released by proteinase-K digestion and quantitated using primers towards EBV oriLyt. The relative amount of encapsidated DNA was calculated using the standard curve method. Z, ZEBRA.

In the previous experiment (Fig 4), we demonstrated that disrupting expression of any of the late gene regulators reduced the level of late transcripts without affecting expression of early genes or amplification of the viral genome. To determine whether such reduction in late transcripts impacts the amount of virus particles released, we purified DNA encapsidated in virions from culture medium of the same samples that were used to generate the data in Fig 4. Quantitative PCR was employed to assess the amount of extracellular virion-protected DNA. The level of extracellular viral DNA detected in the culture medium of 2089 cells transfected with a ZEBRA expression vector was approximately 150-fold higher relative to cells transfected with a control plasmid. Co-transfection of ZEBRA plus siBcRF1, siBDLF4, siBFRF2, siBVLF1, siBDLF3.5, or siBGLF3 reduced the level of extracellular encapsidated viral DNA to 16%, 7.8%, 13%, 1.4%, 23.8%, and 6.9% compared to ZEBRA alone, respectively (Fig 5). In conclusion, while none of the siRNAs diminished the level of intracellular EBV DNA, all siRNAs significantly reduced the level of encapsidated viral DNA released from lytic infected cells. These results suggest that knockdown of late gene regulators affects expression of late genes and subsequent events but has no effect on early events and viral DNA replication.

### BcRF1 is required for the BGLF3-dependent late genes but is dispensable for transcription of the BGLF3-independent late genes

**Fig 6:**
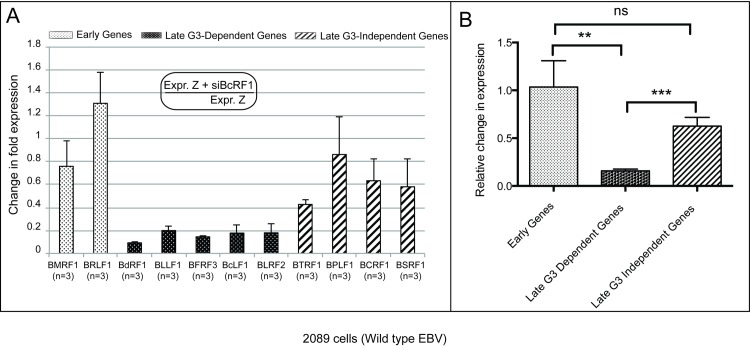
Expression of the four late BGLF3-independent genes does not require BcRF1, the EBV-encoded TBP-like protein. A) The effect of silencing expression of BcRF1 on the level of eleven lytic transcripts was determined by RT-qPCR. Two of these transcripts are expressed early (BRLF1 and BMRF1); nine transcripts are expressed late. Of the nine late transcripts, five are BGLF3-dependent (BdRF1, BLLF1, BFRF3, BcLF1, BLRF2) and four are BGLF3-independent (BTRF1, BPLF1, BCRF1, and BSRF1). Expression of these genes was determined using RT-qPCR of mRNA purified from 2089 cells transfected with ZEBRA or ZEBRA plus siBcRF1. The relative abundance of each transcript was calculated using the *ΔΔC*_*T*_ method. The fold-change in expression of each transcript was determined by dividing the relative abundance of each transcript in the absence and presence of siBcRF1. Letter (n) represents the number of biological replicates. B) A graph illustrating combined changes in expression among the three groups of EBV lytic genes as a result of silencing BcRF1. Asterisks denote statistically significant differences; ns, marks non-statistically significant difference; G3, BGLF3, and Z, ZEBRA.

BcRF1 is a TATA box-binding protein that was previously reported to be required for efficient expression of late gene transcripts in the EBV lytic cycle [13]. Expression of the viral late genes BcLF1, BDLF1 and BLLF1, all of which encode virion structural proteins, was attenuated in the absence of BcRF1 [13]. To determine the effect of BcRF1 on the newly identified BGLF3-independent late genes, we induced lytic infection in 2089 cells by transfecting ZEBRA alone or ZEBRA plus siRNA against BcRF1. Fig 6A shows the level of transcriptional expression of each gene in the absence of BcRF1 compared to levels measured from samples expressing BcRF1. When BcRF1 was knocked down, the early genes BMRF1 and BRLF1 were not significantly affected: BMRF1 was reduced by 1.3-fold (24%) while BRLF1 increased by 1.3-fold (25%). However, the levels of the late BcLF1 and BLLF1 transcripts significantly decreased by 5.6-fold (82%) and 5.0-fold (80%), respectively, compared to the levels detected in samples without siBcRF1 (Fig 6A). Three additional BGLF3-dependent late genes were also significantly impacted; BdRF1, BFRF3 and BLRF2 decreased 10.8-fold (91%), 5.8-fold (83%), and 5.6-fold (82%), respectively, compared to samples not treated with siBcRF1. When fold-changes of early and BGLF3-dependent late genes were averaged and statistically analyzed as a group, the difference between early and late transcripts was significant (*p* = 0.002) (Fig 6B).

The BGLF3-independent subset of late genes was only modestly affected by knockdown of BcRF1; BPLF1, BCRF1 and BSRF1 were reduced by 1.2-fold (14%), 1.6-fold (37%), and 1.7-fold (42%), respectively. Due to the co-terminal nature of the BcRF1 and BTRF1 transcripts, siBcRF1 reduced the level of the BTRF1 transcript by 2.3-fold (57%). Unlike the effect of siBcRF1 on expression of BGLF3-dependent late genes, changes in expression of the BGLF3-independent late genes, excluding BTRF1, were not statistically significant among biological replicates (S1 Table). Thus, the expression pattern of the BGLF3-independent late genes in the presence of siBcRF1 was similar to early genes and different than late genes encoding structural proteins (Fig 6A and S1 Table). The average fold-change between BGLF3-dependent late and BGLF3-independent late genes upon exposure to siBcRF1 is significant (*p* = 0.0007), whereas the change between early and BGLF3-independent late genes is not significant (*p* = 0.13) (Fig 6B). These results demonstrate that while BcRF1 is, in fact, necessary for the expression of a number of late genes, the BGLF3-independent subset of late genes do not require the virally encoded TBP.

Similar findings showing that vTBP is dispensable for expression of BCRF1 (vIL10) were also observed in EBV-infected HH514-16 Burkitt lymphoma cells (S6 Fig). First, we examined whether BCRF1 is expressed with late kinetics by treating ZEBRA-transfected HH514-16 cells with two concentrations of PAA, 0.3 and 0.4 mM. We found that PAA treatment had no effect on synthesis of BMRF1 mRNA and protein relative to untreated cells (S6A and S6D Fig) but markedly reduced expression of the two late genes, BFRF3 (S6B and S6D Fig) and BCRF1 (S6C Fig). Second, we knocked down the late gene regulator vTBP and assessed expression of BMRF1, BFRF3, and BCRF1 as examples of early, BGLF3-dependent late and BGLF3-independent late genes, respectively. We found that expression of BMRF1 and BCRF1 was not affected by siBcRF1 relative to cells transfected with ZEBRA alone, yet expression of BFRF3 was markedly reduced. These results suggest that BCRF1 is a late gene that is expressed in the absence of vTBP BcRF1, one of the main components of the viral pre-initiation complex.

### Absence of late gene regulators BDLF4, BFRF2, BVLF1 and BDLF3.5 does not affect accumulation of the BGLF3-independent late transcripts

Four EBV lytic proteins (BDLF4, BFRF2, BVLF1 and BDLF3.5) in addition to the viral BcRF1 function as regulators of transcription of EBV late genes ([11, 12] and Fig 4). In order to determine if these regulators also impact expression of the BGLF3-independent subset of late genes, we activated the lytic cycle in 2089 cells by transfecting ZEBRA along with siRNA against each of the four late gene regulators. RNA was purified and expression of viral genes was studied using RT-qPCR.

**Fig 7:**
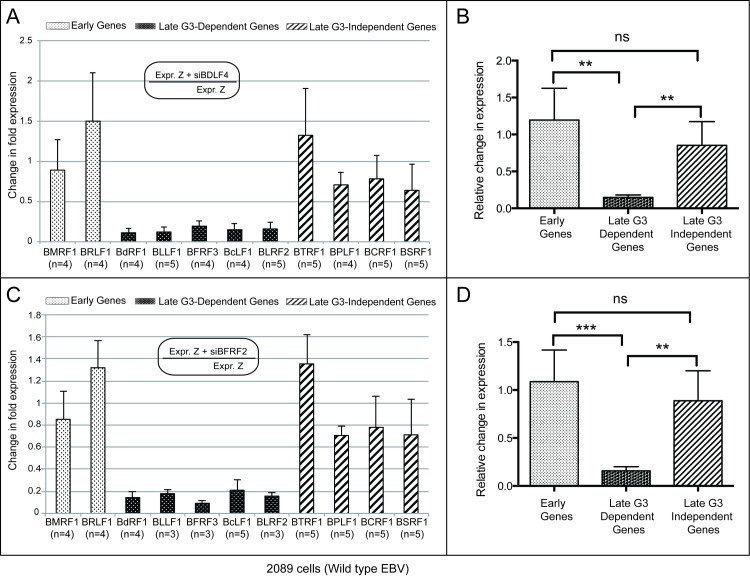
The late gene regulators BDLF4 and BFRF2 are dispensable for expression of BGLF3-independent late genes. The bar graph depicts the effect of silencing expression of BDLF4 (panel A) and BFRF2 (panel C) on the level of eleven transcripts that represent early, late BGLF3-dependent and late BGLF3-independent lytic genes. The experiments were performed in 2089 cells transfected with empty vector, ZEBRA or ZEBRA plus the indicated siRNA. The cells were harvested 48h after transfection. The data were compiled from replicate experiments indicated for each transcript by (n). The bar graphs in panels (B) and (D) illustrate combined changes in fold expression among the three subclasses of lytic genes as a result of transfecting siBDLF4 or siBFRF2. Asterisks denote statistically significant differences; ns, mark non-statistically significant difference; G3, BGLF3, and Z, ZEBRA.

We investigated the effect of siBDLF4 on expression of early, BGLF3-dependent late, and BGLF3-independent late genes. Knockdown of BDLF4 did not reduce transcription of early genes; on the contrary, the level of the BRLF1 early transcript slightly increased compared to samples not treated with siBDLF4 (Fig 7A). Significant decrease in expression of the BGLF3-dependent late transcripts, ranging from 5.1-fold (81%) to 9-fold (89%), was detected in cells transfected with ZEBRA plus siBDLF4 relative to cells solely transfected with ZEBRA (Fig 7A and S1 Table). However, expression of the BGLF3-independent late gene transcripts was not significantly altered in the absence of BDLF4 (S1 Table). The level of the late BTRF1 transcript increased by 1.3-fold (32%) relative to cells transfected with ZEBRA alone, whereas the level of the three other BGLF3-independent late transcripts, BPLF1, BCRF1 and BSRF1, decreased by 1.4-fold (29%), 1.3-fold (22%), and 1.5-fold (35%), respectively (Fig 7A).

**Fig 8:**
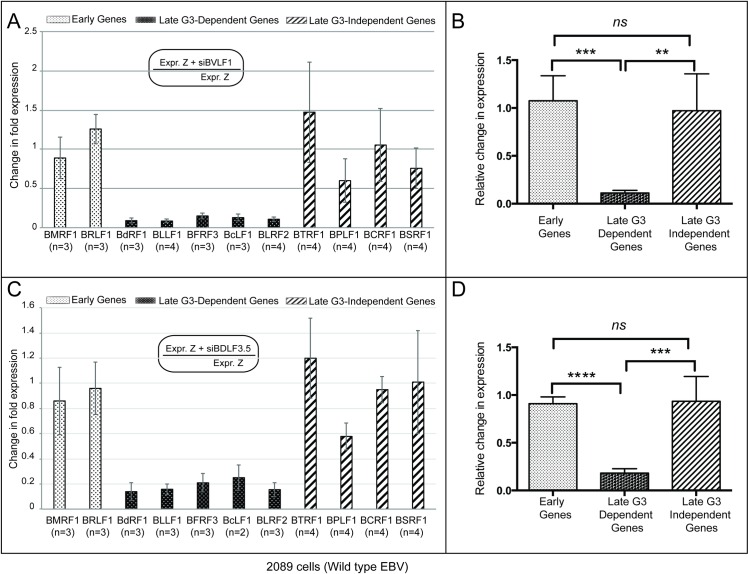
BGLF3-independent late genes are expressed in the absence of BVLF1 and BDLF3.5. A and C) The bar graphs depict the effect of silencing BVLF1 and BDLF3.5 on expression of three groups of lytic genes: early, late BGLF3-dependent and late BGLF3-indpendent. Expression of these genes was determined using RT-qPCR of total RNA purified from 2089 cells transfected with ZEBRA expression vector in the absence and presence of the indicated siRNAs. The data was assembled from replicate experiments; the number of experiments is denoted for each transcript by letter (n). B and D are graphs that illustrate collective changes in expression among the three groups of EBV lytic genes as a result of knocking down BVLF1 or BDLF3.5. Asterisks denote statistically significant differences; ns, mark non-statistically significant difference; G3, BGLF3, and Z, ZEBRA.

Similarly, silencing BFRF2, BVLFI, and BDLF3.5 did not markedly affect accumulation of early or BGLF3-independent late genes but significantly reduced expression of BGLF3-dependent late genes (Figs 7C, 8A and 8C). A marginal effect of all three siRNAs on the level of early transcripts was observed, ranging from a 1.2-fold (15%) decrease to a 1.3-fold (32%) increase relative to expression in cells transfected with ZEBRA alone. Likewise, knockdown of the three late gene regulators, BFRF2, BVLFI, and BDLF3.5 only modestly changed the level of expression of the BGLF3-independent late genes resulting in 1.2-fold (21%) to 1.5-fold (48%) increase in the amount of BTRF1 mRNA; 1.4-fold (29%) to 1.7-fold (40%) decrease in the level of BPLF1 mRNA, and no-change to 1.3-fold (22%) and 1.4-fold (29%) reduction in the levels of BCRF1 and BSRF1 transcripts, respectively. In contrast, transfection of siBFRF2, siBVLF1, or siBDLF3.5 resulted in significant reduction in expression of the BGLF3-dependent late genes that ranged from 5.6-fold (82%) to 10.9-fold (91%), 6.6-fold (85%) to 11.7-fold (91%), and 4.8-fold (79%) to 7.2-fold (86%), respectively (S1 Table).

A statistical approach averaging fold-changes in expression of the three categories of lytic genes revealed similar outcomes among the four siRNAs targeting late gene regulators (Figs 7B, 7D, 8B and 8D). Variations in averaged fold-changes indicated that differences in expression between early and BGLF3-dependent late genes were statistically significant for each of the used siRNAs (siBDLF4 *p =* 0.0014, siBFRF2 *p =* 0.0008, siBVLF1 *p =* 0.0002, and siBDLF3.5 *p <* 0.0001). Differences in the averaged fold-changes between BGLF3-dependent and BGLF3-independent late genes were statistically significant (siBDLF4 *p =* 0.0016, siBFRF2 *p =* 0.0012, siBVLF1 *p =* 0.0014, and siBDLF3.5 *p =* 0.0004). However, no statistical significance was observed when comparing the averaged fold-changes of early and BGLF3-independent late genes. These results reinforce the conclusion that the pattern of expression of the BGLF3-independent late genes is similar to early genes and is independent of the action of the conventional late gene regulators.

### BGLF4 is essential for transcription of the BGLF3-independent and BGLF3-dependent late genes

**Fig 9:**
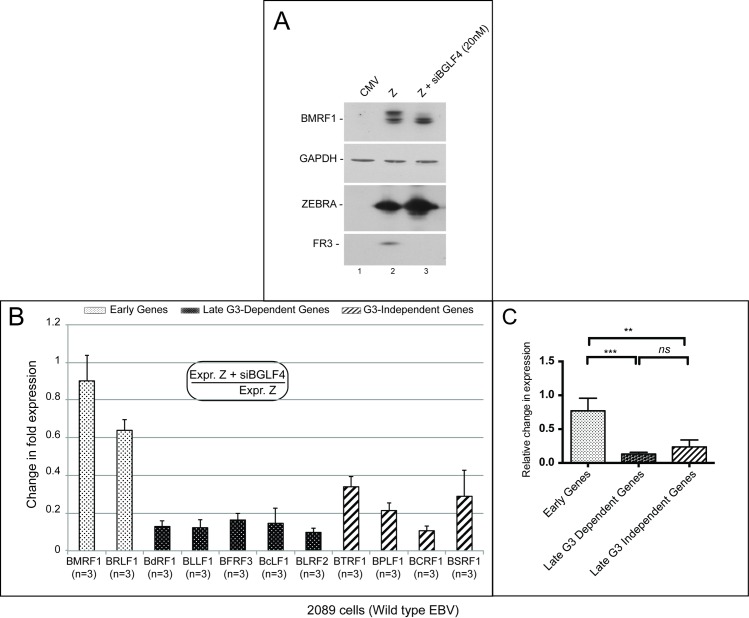
BGLF4 regulates expression of the BGLF3-independent subclass of late genes. A) Immunoblot demonstrating the effect of silencing expression of BGLF4 on the phosphorylation of BMRF1 and the expression of the late viral capsid FR3 protein. B) The bar graph illustrates the effect of silencing BGLF4 on the transcript level of early, BGLF3-dependent and BGLF3-independent late genes. The data were compiled from replicate experiments indicated for each transcript by (n). C) The bar graph demonstrates the combined changes in fold expression among the three subclasses of lytic genes as a result of transfecting siBGLF4. Asterisks denote statistically significant differences; ns, mark non-statistically significant difference; G3, BGLF3, and Z, ZEBRA.

Recently we showed that BGLF4 plays an indispensable role in regulation of late gene expression [10]. To determine whether knockdown of BGLF4 disrupts expression of the BGLF3-independent late genes, we transfected 2089 cells with empty vector, or ZEBRA expression vector in the absence and presence of siRNA to BGLF4. We used Western blot analysis to assess expression of BMRF1 as a marker for induction of the lytic cycle by ZEBRA. BMRF1 is also a bona fide substrate of BGLF4. Silencing expression of the BGLF4 kinase abolished the hyperphosphorylated form of BMRF1 as well as expression of the late BFRF3 gene encoding the small viral capsid protein (Fig 9A). These results confirm our previously published findings showing that BGLF4 is necessary for late gene expression.

Using RT-qPCR we found that silencing expression of BGLF4 marginally reduced the level of the two early transcripts, BMRF1 and BRLF1, by 1.1- and 1.5-fold but markedly reduced the transcript level of both the BGLF3-dependent and the BGLF3-independent subclasses of late genes (Fig 9B and 9C). Our findings demonstrate that the viral BGLF4 protein kinase is the only known late gene regulator necessary for expression of the BGLF3-independent late genes.

### Rta associates with the promoters of two BGLF3-independent genes during the late phase of the EBV lytic cycle

**Fig 10:**
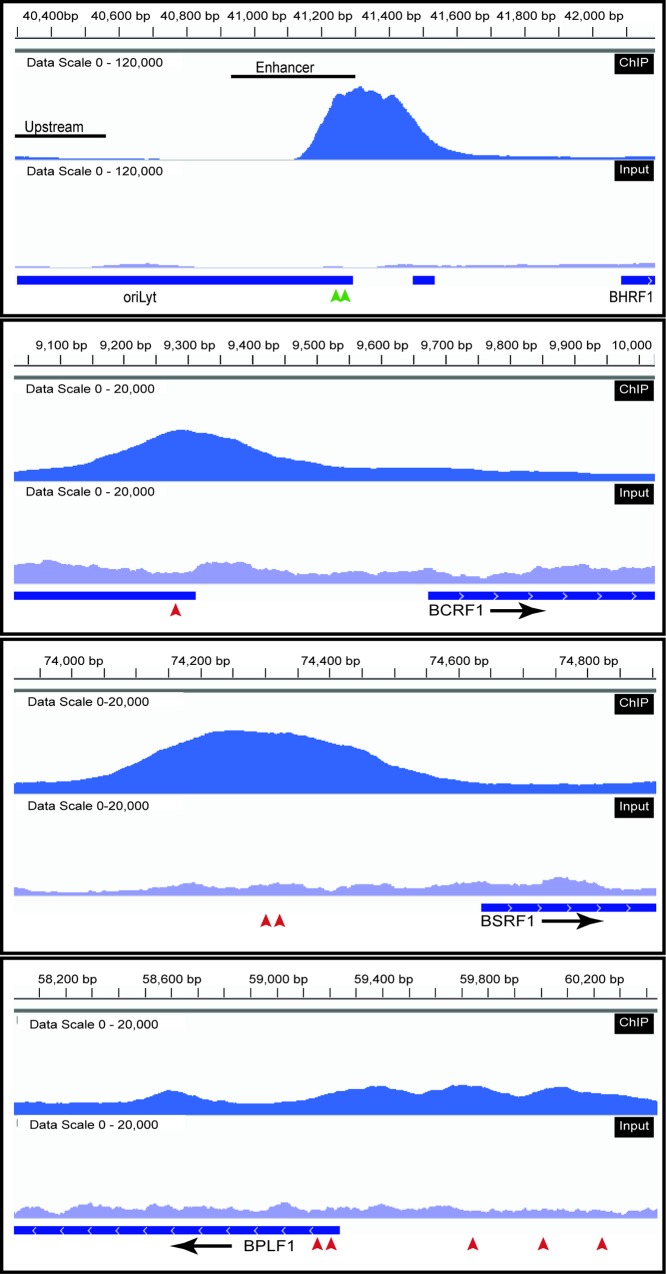
ChIP-seq assessing binding of Rta to the BGLF3-independent late gene promoters. Graphs generated by the Integrative Genomics Viewer (IGV) visualization tool [81, 82] shows four Rta ChIP (dark blue) and Input (light blue) tracks of oriLyt, and the promoter regions of BCRF1, BSRF1 and BPLF1. The data set range for both ChIP and input was 120,000 for oriLyt and 20,000 for BCRF1, BSRF1 and BPLF1.

**Fig 11:**
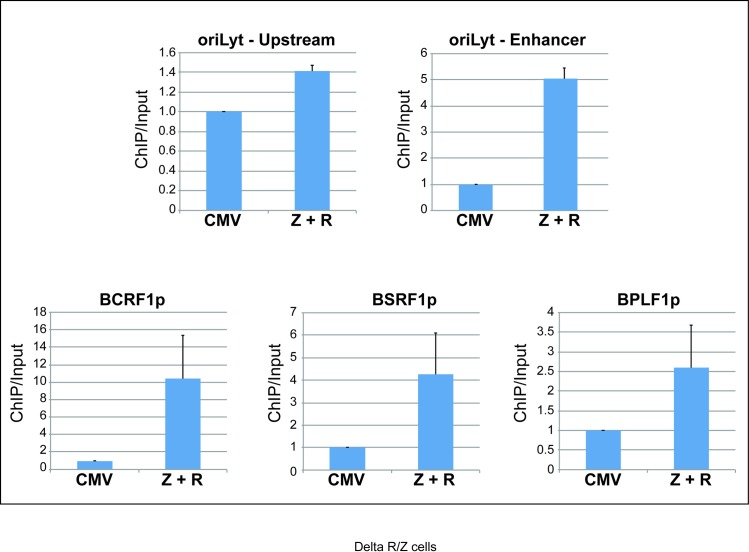
Rta associates with the promoters of the late BGLF3-independent BCRF1, BSRF1 and BPLF1 genes. Chromatin immunoprecipitation examining the level of viral DNA co-immunoprecipitated from delta Rta/ZEBRA cells using Rta antibody. Real time PCR was used to compare association of Rta with the promoters of three BGLF3-independent late genes, BCRF1, BSRF1 and BPLF1. The upstream and the enhancer regions of oriLyt were used as negative and positive controls, respectively, for Rta binding. Fold association of Rta with EBV DNA was corrected for the amount of input DNA. Z, ZEBRA; R, Rta, and oriLyt, the origin of EBV lytic replication.

In an attempt to understand the mechanism that regulates expression of the BGLF3-independent late genes, we searched the primary sequence of BTRF1, BPLF1, BSRF1, and BCRF1 promoters for common potential binding sites of viral and cellular transcription factors. We found that all four BGLF3-independent late promoters contained putative Rta response elements (RREs) that conform to the GNCC(N)_9_GGNG consensus sequence [34, 35]. To determine whether Rta binds to these RREs *in vivo*, we performed chromatin immunoprecipitation using a polyclonal antibody against Rta in delta Rta/ZEBRA cells (293 cells harboring EBV-bacmid with deletions in the genes encoding the lytic cycle activators, Rta and ZEBRA). Ectopic expression of both ZEBRA and Rta in delta Rta/ZEBRA cells is necessary to induce the lytic cycle and to activate viral DNA replication. The cells were transfected with CMV (empty vector), or Rta plus ZEBRA, and harvested after 48h. Rta occupancy was assessed using next generation DNA sequencing of the chromatin-immunoprecipitated DNA. Among the four BGLF3-independent late genes, the promoters of two genes, BCRF1 and BSRF1, exhibited Rta binding peaks that met our criteria for significance (see methods). Three Rta binding peaks were observed upstream of the BPLF1 gene that coincided with predicted Rta binding sites; however, none of the three peaks showed significant binding of Rta when compared to input samples (Fig 10). No obvious peaks were detected upstream of the BTRF1 gene (S7 Fig). To further confirm association of Rta with the BGLF3-independent late promoters, we repeated the ChIP experiment using quantitative PCR to determine association of Rta with the promoters of BCRF1, BSRF1, and BPLF1 (Fig 11). As a control, we assessed binding of Rta to the upstream and enhancer regions in the origin of lytic replication (oriLyt). Previous reports, and our current ChIP-seq data (Fig 10) demonstrated that Rta binds to two RREs present in the enhancer element but does not bind to the upstream region of oriLyt [36–38]. Relative association of Rta with BCRF1p, BSRF1p, BPLF1p and the enhancer region of oriLyt increased by 14-, 4-, 2.5- and 5- fold, respectively, in cells transfected with Rta plus ZEBRA relative to cells transfected with empty vector (Fig 11). However, similar to the ChIP-seq data, Rta association to the upstream region of oriLyt was low, 1.4-fold higher in cells transfected with ZEBRA and Rta relative to empty vector (Fig 11). These results demonstrate that Rta specifically associates with at least two of the four BGLF3-independent late promoters *in vivo*.

In addition to the BGLF3-independent late genes, our ChIP-seq analysis revealed that Rta selectively binds to the promoter region of six other late genes, namely BORF1 (triplex capsid protein), BLRF1 (glycoprotein N), BLRF2 (tegument protein), BBRF3 (glycoprotein M), BGLF2 (tegument protein), and BXRF1 (homolog of HHV1 UL24, a nuclear egress protein). Relative association of Rta with these promoters ranged from 3.1- to 7.7-fold (Table 1). In addition, we observed several Rta binding peaks upstream to known Rta-responsive early genes, e.g. BHRF1, BMRF1, BMRF2, SM, and BGLF3 (Table 1).

**Table 1:** Rta ChIP-seq peaks detected during the late phase of the EBV lytic cycle.

Peak #	Location	Closest ORF within 1kb	Distance to nearest ORF	Peak Mid Point	Fold Change	Distance to RTA predicted site	Kinetics
1	Promoter (BCRF1)	BCRF1	383	9292	3.12	20	Late
2	Promoter (BHRF1)	BHRF1	159	41315	86.58	47	Early
3	Exon (BFLF2)			44424	4.11	451	
4	Promoter (BFLF2)	BFLF2	339	44986	4.08	111	Early
5	Promoter (BFLF2)	BFLF2	769	45416	4.48	541	Early
6	Promoter-TSS (BFRF1)	BFRF1	93	46143	8.93	29	Early
7	Promoter-TSS (BFRF2)	BFRF2	89	47431	4.31	199	Early
8	Exon (BFRF3)			49742	8.03	102	
9	Exon (BPLF1)			50306	4.28	127	
10	Exon (BPLF1)			51204	5.27	276	
12	Promoter (BORF1)	BORF1	647	62303	3.43	413	Late
13	Promoter (BORF1)	BORF1	105	62845	7.65	129	Late
14	Promoter (BORF2)	BORF2	321	63798	8.89	336	Early
15	Exon (BORF2)			64675	4.71	206	Early
16	Promoter (BaRF1)	BaRF1	199	66413	4.82	7	Early
17	Promoter (BMRF1)	BMRF1	342	67269	12.85	150	Early
18	Promoter (BMRF2)	BMRF2	80	68750	11.71	106	Early
19	Exon SM			71658	6.84	85	Early
20	Promoter (SM)	SM	422	72422	13.37	2	Early
21	Promoter (BSRF1)	BSRF1	332	74261	6.79	53	Late
22	Promoter (BLRF1)	BLRF1	776	75483	3.32	310	Late
23	Promoter (BLRF2)	BLRF2	282	76355	5.67	12	Late
24	Exon BLLF2			77548	5.59	463	
25	Promoter (EBNA-3A.1)	EBNA-3A	884	79071	12.29	1986	Latent
26	Exon (EBNA-3C)			88026	14.57	3	
27	Promoter (BKRF3)	BKRF3	352	97713	6.09	13	Early
28	Promoter (BBRF3)	BBRF3	778	106071	4.93	0	Late
29	Promoter (BBLF2/BBLF3)	BBLF2/BBLF3	146	106896	7.25	487	Early
30	Promoter (BBLF2/BBLF3)	BBLF2/BBLF3	778	107528	4.23	145	Early
31	Promoter (BBLF1)	BBLF1	879	109565	4.99	79	Early
32	Promoter (BGLF5)	BGLF5	14	110067	4.63	134	Early
33	Promoter (BGLF4)	BGLF4	148	111474	6.2	689	Early
34	Promoter (BGLF3.5)	BGLF3.5	430	112096	7.37	67	Early
35	Promoter (BGLF3)	BGLF3	295	112946	17.08	347	Early
36	Promoter (BGLF2)	BGLF2	110	114695	6.77	14	Late
37	Promoter (BXRF1)	BXRF1	497	132074	5.94	269	Late
38	Exon BdRF1/BVRF2			136643	12.77	555	
39	Exon (BALF4)			157560	7.42	140	
40	Promoter (BALF2)	BALF2	304	164616	8.21	260	Early
41	Promoter (BNLF2a)	BNLF2a	992	168020	5.96	926	Early

ChIP-seq was performed with antibodies to Rta 48 hours after transfection of Rta and ZEBRA expression vectors into delta Rta/ZEBRA cells. The promoter of a gene was hypothetically defined as a 1 kb region upstream of the open reading frame. Peaks present outside of the 1 kb range were indicated by their annotated location in the EBV genome. The distance to the nearest open reading frame was calculated from the mid-point of the ChIP-seq peak to the nearest open reading frame. The distance to an Rta predicted site was calculated as the distance from the mid-point of the Rta ChIP peak to the mid point of the Rta consensus binding sequence. ChIP-seq peaks with a fold change less than 3-fold relative to their corresponding input peaks were excluded.

In conclusion, our findings demonstrate the presence of at least two independent mechanisms for transcription of late genes. One mechanism, involving the conventional late gene regulators, is responsible for transcription of BGLF3-dependent late genes encoding structural proteins. The other mechanism, involving Rta and the BGLF4 protein kinase, regulates transcription of four BGLF3-independent late genes two of which encode viral immunoevasins.

## Discussion

The novel findings reported here will enhance and expand the current understanding of the mechanisms that regulate expression of herpesvirus late genes. Seven late gene regulators mediate transcription of EBV late genes, these include: the viral protein kinase BGLF4 and six proteins that form a distinct viral pre-initiation complex (vPIC) on late promoters. Whether the functions of these late gene regulators overlap and whether the same set of late gene regulators controls transcription of all late genes are questions that have not been addressed previously. In this report, we provide novel answers to these questions: 1) we demonstrate that all the components of vPIC are essential and functionally non-redundant in expression of EBV structural proteins and subsequent formation of virus particles (Figs 4 and 5). 2) We identify a subset of late genes that are transcribed in the absence of vPIC (Figs 1–3 and 6–8). These vPIC-independent late genes include genes that encode two viral immunoevasins, vIL10 and vDUB. 3) We show that the BGLF4 protein kinase is the only late gene regulator that is necessary for transcription of vPIC-dependent and vPIC–independent late genes (Fig 9). 4) We demonstrate that the Rta transcription activator selectively binds to eight late promoters including the promoters of two vPIC-independent late genes, BCRF1 (vIL10) and BSRF1 (Figs 10 and 11 and Table 1). Therefore, Rta is likely to regulate transcription of a subset of late genes.

### Differential regulation of late gene expression

The current model for regulation of late gene expression suggests that all late genes are regulated by a single common mechanism involving viral DNA replication and the functions of seven late gene regulators [10, 11, 13, 15, 16, 18–21, 39–42]. Recent identification of these key regulators of late gene expression prompted us to ask whether there are different subclasses of late genes depending on their mechanism of expression. For example, each subclass of late genes might require the activity of a unique set of late gene regulators. To address this question we studied the EBV transcriptome during the late phase of the viral lytic cycle under conditions in which expression of BGLF3, a component of vPIC, was silenced (Fig 1). This set of experiments led to the identification of four BGLF3-independent genes that were expressed with true late kinetics (Figs 1–3 and S2). To determine the importance of the other components of vPIC in expression of the BGLF3-independent late genes, we generated specific siRNAs to BcRF1 (vTBP), BDLF4, BFRF2, BDLF3.5, and BVLF1. Knockdown of each of these late gene regulators was dispensable for expression of the four BGLF3-independent late genes (Figs 6–8 and S6). On the contrary, elimination of any one of the late gene regulators significantly reduced the level of several late transcripts encoding structural proteins, an effect which was manifest as a decreased level of extracellular encapsidated viral DNA (Fig 5). siRNAs to the late gene regulators did not significantly reduce expression of early genes or the level of intracellular viral DNA, a measure of the process of viral genome amplification (Figs 4–8 and S1 Table). These findings demonstrate the presence of at least two subsets of late genes, vPIC-dependent and vPIC-independent. In addition, our results establish the essential and non-redundant role of each of the conventional late gene regulators in transcription of late genes encoding the major and minor capsid proteins (BcLF1 and BFRF3), the scaffold protein (BdRF1), and the major glycoprotein gp350 (BLLF1).

### Role of vTBP in regulation of late gene expression

At the mechanistic level we are just beginning to understand some of the functions of the late gene regulators. BcRF1 and its orthologs function as TATA box binding proteins that recognize late promoters and recruit RNA polymerase II to late promoters through direct protein-protein interactions [11, 16, 18]. Based on our findings, BcRF1 is dispensable for transcription of the BGLF3-independent late genes (Fig 6). BcRF1 recognizes late promoters that contain a distinct TATT element with a thymidine at position 4 [13, 17]. However, this feature is not preserved in all late genes. At least six late gene promoters contain a canonical TATA box: BCRF1, BBRF3, BLRF1, BRRF2, BDLF3, and BXLF2. Whether BcRF1 binds to these promoters is still unknown. Among the BGLF3-independent late genes, the promoter of the BCRF1 (vIL10) gene harbors a canonical TATA box while the promoters of BPLF1 and BSRF1 contain the unconventional TATT element. No TATA box element has been assigned for the BTRF1 gene. Knockdown of BcRF1 did not compromise transcription from the BPLF1 or the BSRF1 late genes suggesting that the TATT sequence in the promoters of these two genes is likely to be recognized by cellular or viral proteins other than the vTBP (Fig 6).

### BGLF4 is required for transcription of vPIC-dependent and -independent late genes

The BGLF4 protein kinase is the only late gene regulator essential for transcription of the two subclasses of late genes. BGLF4 is likely to phosphorylate substrates that regulate transcription of both groups of late genes. For example, BGLF4 might phosphorylate a component of the general transcription machinery or one of the late gene regulators to promote recruitment of RNA polymerase II to late promoters or to facilitate the transcription elongation phase during synthesis of late mRNAs. BGLF4 is also known to phosphorylate proteins involved in activation of DNA damage response, e.g. the histone acetyltransferase TIP60, which is involved in chromatin remodeling [43]. Activation of late gene expression may be linked to the DNA damage response pathway through the capacity of BGLF4 to phosphorylate TIP60 which then alters chromatin structure around late promoters [44, 45].

Transcription of the vPIC-independent late genes is likely to be regulated by viral proteins that could be subject to phosphorylation by BGLF4. One potential candidate is Rta. Our chromatin immunoprecipitation data indicate that Rta strongly binds to the promoters of two vPIC-independent late genes, BCRF1 and BSRF1. Binding of Rta to these vPIC-independent late promoters suggests that Rta might function as a late gene activator. Rta is a phosphoprotein; phosphorylation occurs late during the lytic cycle [46, 47]. The kinase that phosphorylates Rta and the Rta phosphorylation sites are yet to be identified. A possible scenario is that Rta becomes phosphorylated by BGLF4 late during lytic infection. This scenario is further supported by findings demonstrating that the kinase activity of BGLF4 is essential for transcription of late genes [10]. Rta and BGLF4 co-localize in viral replication compartments, a locale where BGLF4 might promote phosphorylation of Rta during the late phase [48–50].

### Potential role of Rta in regulating expression of EBV late genes

Our ChIP-seq data (Table 1) shows that Rta also binds upstream of six other late genes: BORF1, BLRF1, BLRF2, BBRF3, BGLF2, and BXRF1, that are dependent of vPIC (Table 1). These results confirm previous reports implicating Rta in regulation of late gene expression. For instance, Rta induces expression of the late BLRF2 gene by directly binding to its promoter [34, 37, 51, 52]. Our ChIP experiments were performed in delta Rta/ZEBRA cells, which require ectopic expression of both Rta and ZEBRA to activate viral genome amplification. Experiments assessing whether the presence of ZEBRA or the onset of viral DNA replication are necessary for the capacity of Rta to bind to late promoters are currently underway. While several cellular transcription factors, such as TBP, TFIIB, TAF4, Sp1, ATF2, and TSG101, mediate Rta transcriptional activity [53–58], the mechanism regulating temporal activation of late genes by Rta has not been elucidated. Our data demonstrate that transcription of BLRF2 is regulated by vPIC (Figs 2 and 6–8). Therefore, Rta might interact with one or more components of the vPIC to activate transcription of the late BLRF2 gene. Such protein-protein interaction might regulate the capacity of Rta to temporally activate transcription of a subset of late genes. Alternatively, phosphorylation of Rta by BGLF4 during the late phase of the lytic cycle might also regulate the temporal role of Rta in activation of a subset of late genes.

### Implications for identifying multiple mechanisms that regulate expression of late genes

Viral infection is often accompanied by triggering of innate immune responses. However, incoming virus particles are usually equipped with a mechanism that subverts the immune response to allow infection to proceed. In the case of EBV, several virally encoded proteins were shown to interfere with host immune defense mechanisms to promote progress of lytic replication and primary infection [59]. Two of these viral immune suppressants are the products of the BCRF1 and the BPLF1 genes, which encode a homolog of human IL-10 (vIL-10) and a viral deubiquitinase (vDUB), respectively [23, 26]. BCRF1 and BPLF1 are expressed in the late phase of the lytic cycle [23, 30, 60, 61]; the mechanism regulating their expression has not been studied. Our findings demonstrate that both genes belong to the vPIC-independent subclass of late genes (Figs 1, 2 and 3). The BCRF1 transcript and the BPLF1 protein are packaged into virus particles and delivered to newly infected cells [23, 62]. Both vIL-10 and vDUB are essential to subvert the immune response during primary infection [23, 26, 27, 63, 64]. vIL10 impairs NK cell-mediated elimination of newly infected B cells; disrupts secretion of antiviral cytokines, and interferes with the antiviral activity of CD4+ effector T cells [26]. BPLF1 counteracts innate antiviral immune responses by inhibiting NF-κB activation. Through its DUB activity, BPLF1 removes K48- and K63-ubiquitin moieties from TRAF6, NEMO, and IκBa and hence blocks the Toll-like receptor-signaling pathway [23]. Lack of vDUB also interferes with establishment of EBV infection and virus production [24, 65–67].

An interesting question is why a subclass of late genes is transcribed by a distinct mechanism relative to other late genes. Considering the fact that two of the vPIC-independent late genes (BCRF1 and BPLF1) encode viral immunoevasins, it is possible that expression of this subclass of late proteins precedes expression of the late antigenic structural proteins to prevent immune recognition of late lytic cells. However, this difference in timing would still be within the late phase of the lytic cycle as both classes of genes are sensitive to inhibition of viral DNA replication (Figs 3 and S2). Alternatively, the presence of distinct mechanisms that regulate transcription of these two subclasses of late genes might be attributed to the level of their expression.

In conclusion, in this study we demonstrate that the mechanism regulating expression of the late immunomodulators vIL10 and vDUB differs from that regulating synthesis of late structural proteins. While transcription of the late genes encoding structural proteins is dependent on the six components of vPIC, transcription of BCRF1, BPLF1, BSRF1, and BTRF1 is vPIC independent. Delineating mechanisms regulating expression of the vPIC-independent late genes and selectively inhibiting their expression has the potential to promote immune recognition of EBV during the late phase of the productive lytic cycle and during de novo infection. The studies described here might have relevance for vaccine development.

## Materials and Methods

### Expression vectors

The ZEBRA and Rta protein expression vectors were prepared as previously described [68, 69]. Expression vectors of BGLF3, BVLF1, BDLF4, and BDLF3.5 were cloned into the eukaryotic pCMV6-Entry vector using the SgfI and MluI unique sites (Origene). Expression vectors of the two late gene regulators, BcRF1 and BFRF2, were kind gifts from Dr. Eric Johannsen. The construct expressing the BGLF4 protein kinase protein was a kind gift from Dr. Mei-Ru Chen [70]. siRNA-resistant forms were produced by inserting silent mutations in the region of the late gene regulator mRNA that is recognized by the siRNA. These silent mutations disrupt the complementarity between the siRNA and its target late gene regulator without affecting the amino acid sequence of the protein.

### Cell culture and transfection

2089 cells are 293 human embryonic kidney (HEK) cells stably transfected with a bacmid containing wild-type EBV B95-8 genome [71, 72]. Delta Rta/ZEBRA and delta BMRF1 cells are 293 HEK cells containing EBV-bacmid in which the BZLF1 and BRLF1 genes encoding ZEBRA and Rta or the BMRF1 gene, respectively, were inactivated by insertion of a kanamycin resistance gene. All three types of cells were cultured in Dulbecco’s modified Eagle medium (DMEM) supplemented with 10% fetal bovine serum (FBS), and penicillin-streptomycin at 50 units/ml. Hygromycin B (Calbiochem) 100 μg/ml was added to the medium to select for 293 cells containing the EBV bacmid. Transfection of eukaryotic plasmids was performed in 25 cm^2^ flasks using Lipofectamine 2000 (Invitrogen) following the manufacturer’s protocol. Transfections were carried out in OPTI-MEM medium.

HH514-16 cells were derived from the P3J-HR1K Burkitt lymphoma cell line [73]. The cells were cultured in RPMI 1640 medium containing 10% fetal bovine serum and antibiotics. To induce the lytic cycle, HH514-16 cells were transfected with 4μg empty vector (CMV) or ZEBRA expression vector using Ingenio nucleofection reagent according to the manufacturer’s protocol (Mirus). Cells were incubated at 37°C in 5% CO_2_ incubator and harvested 48 h after transfection.

### Knockdown of late gene regulators

For each late gene regulator, two siRNAs were designed and separately tested for their efficiencies to disrupt expression of the late FR3 protein without affecting expression of the early BMRF1 protein (S3 Fig). The selected siRNAs were then tested for their capacities to knockdown the levels of their corresponding late gene regulator transcripts (Table 2 and S4 Fig). The specificities of these siRNAs were also examined by generating siRNA-resistant forms of each late gene regulator (S5 Fig) and [10].

**Table 2:** Sequences of siRNAs used to silence expression of individual components of vPIC.

*siRNA*	*Sequence*
siBcRF1-A	AGAAGUGGAACUUGAGUCUGGCCUC
siBDLF4-A	AACAGCAUCAGGCAGCAAGGGAACA
siBFRF2-A	CUCAUUUCCACAUAGCUCAGAUCCA
siBVLF1-B	AACACUUAGGGUCAGCAGCUUGGUC
siBDLF3.5-A	AAUAGUGCCUCGCUUCUUAUCCUGU

### Protein detection by Western blot

Cells were re-suspended in sodium dodecyl sulfate (SDS) sample buffer; 10^6^ cells/sample were loaded onto 10% SDS-polyacrylamide gels and electrophoresed. The separated proteins were transferred to nitrocellulose membranes (Bio-Rad) and the membranes were blotted with specific antibodies to cellular and viral proteins. The ZEBRA and BFRF3 antibodies were described previously [38]. The EA-D (BMRF1) monoclonal antibody (R3.1) was provided by G. Pearson [74]. GAPDH and FLAG-tagged BGLF3 were detected using mouse monoclonal antibodies (Sigma Aldrich). Antigen-antibody complexes were detected by autoradiography using ^125^I-protein A or chemiluminescence (GE Healthcare Life Sciences).

### Quantitative RT-PCR

RNA was prepared from cells using the Qia-shredder and the RNeasy Plus products from Qiagen. The concentration of RNA in each sample was determined by measuring the optical density at 260 nm. Viral transcript levels were assessed from 100 ng of total RNA using iTaq Universal SYBR Green One-Step Kit (Bio-Rad) in a total volume of 25 μl. The level of GAPDH RNA was measured to normalize for the total amount of RNA. Each sample was analyzed in triplicate; the fold change in expression was calculated using the ΔΔC_T_ formula. The efficiency of the primers used in the RT-qPCR was determined against 10-fold increasing concentrations of viral DNA. The sequences of the primers are provided in Table 3.

**Table 3:** Sequences of Primers used in real time PCR assays.

Gene	Forward Primer (5’-3’)	Reverse Primer (5’-3’)
BBLF1	TAA GCT AAT AGC GGC CGA AG	TTG GCA AAA ACA GCA ATT CA
BcLF1	ATA CCG GAG CAC TGT GAA TC	TCC TCC TTG TTG AAA AAT GC
BcRF1	AGG GAC CTG TAA GCG AAA CT	GTG CTC CAG GAG AAG AAG TG
BCRF1	CAG GCC CTG TCA GAA ATG AT	TCC TTT TTC CTG CAG CTT GT
BDLF3.5	AGA GAG AAT TTG GAG AAT TG	CTT GGT CTG ACA TTC CAC
BDLF4	GTG GAT GTG GTC CTC AGT CT	ATC ATA GCG CTT GTT TCT GG
BdRF1	GCT ATC AGG TAA CGC AGG AG	GTT GGT CTG AAG CAG TGT CA
BFRF2	GTA AGC CGC AAT GTT CTC TC	GCT TTC CAA AGT TCA AGC AG
BFRF3	GCC ATA GAC AAG AGG CAG AG	CGG AGG CTG CTA ATA GAT GA
BGLF3	GGG TCT GTG TTT TGA GGT GT	TAT AAA CCG CAG TGG GTA GC
BGLF4	AAA AGA GGT TCA AGG AGA GCT AC	AGT CGT CTG CCA AGA GTT CA
BLLF1	TGC TGA TCC CAA TAC AAC GA	TGG AGA TGG ACT TGG TGT CA
BLRF2	CTC TGA AGC AAC AGG TCC TC	TCC GAA CCT TGT CTT CAA TC
BMRF1	CAA CAC CGC ACT GGA GAG	GCC TGC TTC ACT TTC TTG G
BMRF2	CCC TTC ATT TGG TGC TTT GT	GTT GGC CAG AAA GAG ACA GG
BOLF1	CTG GTG GCC GAC ACT TAT CT	ATA CGG TGA CCA GCC ATC TC
BPLF1	CGG GGT CAC AGA AGA AAC AT	GTC CTG AGA GGA GGC TTG TG
BRLF1	CCA TAC AGG ACA CAA CAC CTC A	ACT CCC GGC TGT AAA TTC CT
BSRF1	TTC AAG CCA ATA GCA TCA CG	CCG CTT ACA GCC AGA GAG AT
BTRF1	AGC CTC CTC GCA ACT TAT GA	CAG GGA TGA GAG CAC AGT CA
BVLF1	GAA TGA CAC CTG GGA TTC AC	GTC AGG GAT AGG TGC AAA AA
oriLyt	TCC TCT TTT TGG GGT CTC TG	CCC TCC TCC TCT CGT TAT CC

### Next generation RNA sequencing and data analysis

Three strand-specific sequencing libraries were generated using total RNA purified from 2089 cells transfected with CMV, ZEBRA, or ZEBRA plus siBGLF3. The libraries were sequenced using the Illumina HiSeq 2500 system. The reads were single-end and 150bp long. The first 6 nucleotides and the last 60 nucleotides in each read were trimmed to remove low quality bases using FASTX toolkit (http://hannonlab.cshl.edu/fastx_toolkit/index.html). Reads were mapped to the human reference genome (hg19) with a known transcriptome index (UCSC Known Gene annotation) using Tophat v2.0.8 [75]. Reads that did not map to the human genome were later mapped to the EBV genome (GenBank accession number NC_007605.1 excluding the B95-5 deletion) with a known transcriptome annotation [76]. ZEBRA and ZEBRA plus siBGLF3 had over 450,000 reads mapping to the EBV genome. We used the Expectation-Maximization (EM) algorithm in RSEM [77] with Bowtie 2 [78] to map and estimate gene expression levels. EBSeq within RSEM pipeline was used to identify differentially expressed genes [79].

### Viral DNA replication assay

Viral DNA was purified from cell pellets as described [10]. Total DNA concentration was determined by measuring absorbance at 260 nm. Viral genome amplification was measured using the iQ SYBR Green Supermix kit (Bio-Rad) and primers targeting oriLyt (Table 2). Relative concentrations of DNA were calculated based on a standard curve of known concentrations of oriLyt DNA. Levels of viral DNA were normalized to a negative control sample transfected with the empty vector CMV.

### Virion assay

Supernatant from transfected cells was collected 48 h after initial transfection and spun twice at 1500 rpm to remove cell debris. The collected supernatant was treated with DNase-I (7 μg/ml) and RNase (7 μg/ml) for 30 min at 37°C in the presence of 3 mM MgCl_2_ and CaCl_2_. This mixture was centrifuged at 77,000 xg for 30 min at 4°C to pellet viral particles. The pellet was resuspended in TE buffer containing 0.5% SDS. Pronase (1.2 mg/ml) was added and the solution was incubated at 60°C for 2 h to digest the viral capsid. Phenol-chloroform extraction was used to remove proteins and the DNA was precipitated using 5M potassium acetate and 2.5 volumes of ethanol. The DNA precipitate was washed with 70% cold ethanol and re-suspended in TE buffer. The level of extracellular viral DNA was determined by qPCR using primers towards the upstream region of oriLyt.

### Chromatin immunoprecipitation (ChIP) and ChIP-seq analysis

Immunoprecipitation of viral DNA was performed by chemically crosslinking DNA-protein complexes formed in 2089 cells using 1% formaldehyde. The cells were incubated for 10 min at 37°C and then washed once in phosphate-buffered saline containing protease inhibitors (Thermo Scientific). Cells were re-suspended in SDS lysis buffer (50 mM Tris-HCl [pH 8.1], 1% SDS, and 10 mM EDTA) and sonicated four times, 10s each, using Sonifier 450 apparatus (Branson). Cell lysates were cleared by centrifugation and the collected supernatants were diluted 10-fold in chromatin immunoprecipitation (ChIP) dilution buffer (16.7 mM Tris-HCl [pH 8.1], 0.01% SDS, 1.1% Triton X-100, 167 mM NaCl, and 1.2 mM EDTA). Rta-associated DNA was immunoprecipitated using a rabbit polyclonal antibody (S2454) generated against the full-length protein [38]. The immune complexes were collected on protein G agarose beads (Millipore). Binding of Rta to viral DNA was assessed by quantitative PCR and by next generation sequencing.

Sequencing of ChIP and Input samples was performed using an Illumina HiSeq 2500 sequencer generating 27 and 40 million reads for the CMV ChIP sample and its input control, respectively, and 22 and 30 million reads for the Rta plus ZEBRA ChIP sample and its input control, respectively. The generated reads were single-end and each read was 76bp long. The first and the last nucleotides for each read were trimmed with fastx-toolkit (http://hannonlab.cshl.edu/fastx_toolkit/index.html) to remove low quality bases. Trimmed reads were mapped to the human reference genome (hg19) using BWA-MEM v0.7.12 [80]. Only reads with mapping quality scores equal or higher than 20 were kept. Those reads that did not map to the human genome were later mapped to the EBV genome (GenBank accession number NC_007605.1) also using BWA-MEM. Peak finding was performed using HOMER [80]. For peak finding, we used transcription factor mode requiring each putative peak to have at least 3-fold normalized tags than input controls. Assigning Rta peaks to viral gene promoters was based on the presence of a peak within 1kb from an open reading frame. Filtering for local and clonal signal was set to off.

## Supporting Information

S1 Fig Visualization of the number of reads mapping to select viral transcripts in the absence and presence of siBGLF3. Graphs generated by the Integrative Genomics Viewer (IGV) visualization tool [81, 82] compare the effect of siBGLF3 on the number of reads mapping to the four BGLF3-independent late transcripts (A to D), three BGLF3-dependent late transcripts (E to G) and the BGLF3 transcript (H). The Bam files used to plot these graphs were obtained from RNA-seq data assessing the effect of siBGLF3 on viral gene expression during the lytic cycle (Fig 1). The data set range is indicated at the top left corner of each graph.
(TIF)

S2 Fig Examining the expression kinetics of the BGLF3-independent late genes in replication defective delta BMRF1 cells. A) Western blot analysis assessing the expression of BMRF1 (early), ZEBRA and BFRF3 (late) in delta BMRF1 cells transfected with empty vector (CMV), ZEBRA (Z), or ZEBRA (Z) plus BMRF1. The extent of intracellular viral DNA replication (V. DNA Repl.) was assessed in the absence and presence of BMRF1. Total DNA was prepared from the same samples and analyzed by qPCR using primers specific to EBV oriLyt. B-H) RT-qPCR measuring the level of seven lytic transcripts: BRLF1 (early), BBLF2/3 (early), BLLF1 (BGLF3-dependent late), BTRF1 (BGLF3-independent late), BSRF1 (BGLF3-independent late), BPLF1 (BGLF3-independent late), and BCRF1 (BGLF3-independent late). Lack of BMRF1, an essential replication protein, abolished expression of the four BGLF3-independent genes.
(TIF)

S3 Fig Examining the effect of silencing expression of BcRF1, BDLF4, BFRF2, BVLF1 and BDLF3.5 on synthesis of the late FR3 protein. Western blot analysis assessing the effect of siRNAs to BcRF1, BDLF4 (panel A), BFRF2 (panel B), BVLF1, and BDLF3.5 (panel C) on expression of the late FR3 protein. The effect of each siRNA was studied at two concentrations, 40 nM and 80 nM. siRNAs marked in bold font were employed in subsequent analyses.
(TIF)

S4 Fig Knockdown of individual late gene regulators reduces their transcript levels. 2089 cells were transfected with empty vector (CMV), ZEBRA (Z) alone, or ZEBRA plus the indicated siRNA. Cells were harvested after 48h and total RNA was purified. The abundance of five lytic transcripts encoding BcRF1, BVLF1, BDLF4, BFRF2, and BDLF3.5 was quantitated using RT-qPCR.
(TIF)

S5 Fig Expression of siRNA-resistant forms of each of the late gene regulators restores synthesis of late transcripts. To assess the specificity of each of the siRNAs generated towards the components of vPIC, we introduced silent mutations that disrupt the ability of each siRNA to bind to its corresponding mRNA. The following four siRNA-resistant forms were generated: rBcRF1, rBVLF1, rBFRF2, and rBDLF4. The experiment was performed in 2089 cells carrying wild type EBV. Panel A shows the capacity of rBcRF1 and rBVLF1 to restore expression of the late FR3 capsid protein in the presence of siBcRF1 and siBVLF1, respectively. Panels B and C demonstrate the capacity of rBFRF2 and rBDLF4 to rescue expression of late genes in the presence of their corresponding siRNA. rBcRF1 and rBFRF2 are HA-tagged and were detected using HA antibody. rBVLF1 and rBDLF4 are FLAG-tagged and were detected using a FLAG antibody.
(TIF)

S6 Fig Expression of the late viral IL10 (BCRF1) is independent of vTBP (BcRF1) in Burkitt lymphoma cells. A-C) RT-qPCR assessing the kinetics of BCRF1 (vIL10) and its dependence on vTBP in HH514-16 Burkitt lymphoma cells. HH514-16 cells were transfected with empty vector (CMV) or ZEBRA (Z) to induce the lytic cycle. ZEBRA-transfected cells were treated with two concentrations (0.3 and 0.4 mM) of phosphonoacetic acid (PAA) or co-transfected with siBcRF1 (50 nM). Panel D shows a Western blot of cell lysates prepared from the same samples analyzed in panels A, B and C. The membrane was immunoblotted with antibodies to BMRF1, GAPDH, ZEBRA (Z), and BFRF3 (FR3).
(TIF)

S7 Fig No Rta binding peaks were detected upstream of the BTRF1 gene. Graphs generated by the Integrative Genomics Viewer (IGV) visualization tool [81, 82] shows Rta ChIP (dark blue) and Input (light blue) tracks of the region upstream of BTRF1 gene.
(TIF)

S1 Table Analysis of RT-qPCR data studying the effect of siRNAs to late gene regulators on expression of select EBV genes. (DOCX)

## References

[1] Epstein MA, Barr YM, Achong BG. Studies with Burkitt's lymphoma. Wistar Inst Symp Monogr. 1965;4:69–82. 5884338

[2] Old LJ, Boyse EA, Oettgen HF, Harven ED, Geering G, Williamson B, et al Precipitating antibody in human serum to an antigen present in cultured burkitt's lymphoma cells. Proc Natl Acad Sci U S A. 1966;56(6):1699–704. PubMed Central PMCID: PMCPMC220158. 16591407 10.1073/pnas.56.6.1699 PMC220158

[3] zur Hausen H, Schulte-Holthausen H, Klein G, Henle W, Henle G, Clifford P, et al EBV DNA in biopsies of Burkitt tumours and anaplastic carcinomas of the nasopharynx. Nature. 1970;228(5276):1056–8. 4320657 10.1038/2281056a0

[4] Weiss LM, Strickler JG, Warnke RA, Purtilo DT, Sklar J. Epstein-Barr viral DNA in tissues of Hodgkin's disease. Am J Pathol. 1987;129(1):86–91. 2821817 PMC1899692

[5] Imai S, Koizumi S, Sugiura M, Tokunaga M, Uemura Y, Yamamoto N, et al Gastric carcinoma: monoclonal epithelial malignant cells expressing Epstein-Barr virus latent infection protein. Proc Natl Acad Sci U S A. 1994;91(19):9131–5. 8090780 10.1073/pnas.91.19.9131 PMC44761

[6] Tokunaga M, Land CE, Uemura Y, Tokudome T, Tanaka S, Sato E. Epstein-Barr virus in gastric carcinoma. Am J Pathol. 1993;143(5):1250–4. PubMed Central PMCID: PMCPMC1887176. 8238241 PMC1887176

[7] Crawford DH, Thomas JA, Janossy G, Sweny P, Fernando ON, Moorhead JF, et al Epstein Barr virus nuclear antigen positive lymphoma after cyclosporin A treatment in patient with renal allograft. Lancet. 1980;1(8182):1355–6. 6104142 10.1016/s0140-6736(80)91800-0

[8] Hanto DW, Sakamoto K, Purtilo DT, Simmons RL, Najarian JS. The Epstein-Barr virus in the pathogenesis of posttransplant lymphoproliferative disorders. Clinical, pathologic, and virologic correlation. Surgery. 1981;90(2):204–13. 6266059

[9] Ziegler JL, Drew WL, Miner RC, Mintz L, Rosenbaum E, Gershow J, et al Outbreak of Burkitt's-like lymphoma in homosexual men. Lancet. 1982;2(8299):631–3. 6125777 10.1016/s0140-6736(82)92740-4

[10] El-Guindy A, Lopez-Giraldez F, Delecluse HJ, McKenzie J, Miller G. A locus encompassing the Epstein-Barr virus bglf4 kinase regulates expression of genes encoding viral structural proteins 2014 [updated Aug; cited 10 8]. e1004307]. Available from: http://www.ncbi.nlm.nih.gov/pubmed/25166506. 10.1371/journal.ppat.1004307 PMC4148442 25166506

[11] Aubry V, Mure F, Mariame B, Deschamps T, Wyrwicz LS, Manet E, et al Epstein-barr virus late gene transcription depends on the assembly of a virus-specific preinitiation complex. J Virol. 2014;88(21):12825–38. PubMed Central PMCID: PMC4248913. 10.1128/JVI.02139-14 25165108 PMC4248913

[12] Watanabe T, Narita Y, Yoshida M, Sato Y, Goshima F, Kimura H, et al Epstein-Barr virus BDLF4 Gene Is Required for Efficient Expression of Viral Late Lytic Genes. J Virol. 2015. 10.1128/JVI.01604-15 PMC4577904 26202235

[13] Gruffat H, Kadjouf F, Mariame B, Manet E. The Epstein-Barr virus BcRF1 gene product is a TBP-like protein with an essential role in late gene expression. J Virol. 2012;86(11):6023–32. Epub 2012/03/30. 10.1128/JVI.00159-12 22457524 PMC3372218

[14] Djavadian R, Chiu YF, Johannsen E. An Epstein-Barr Virus-Encoded Protein Complex Requires an Origin of Lytic Replication In Cis to Mediate Late Gene Transcription. PLoS Pathog. 2016;12(6):e1005718 10.1371/journal.ppat.1005718 27348612 PMC4922670

[15] Wu TT, Park T, Kim H, Tran T, Tong L, Martinez-Guzman D, et al ORF30 and ORF34 are essential for expression of late genes in murine gammaherpesvirus 68. J Virol. 2009;83(5):2265–73. Epub 2008/12/19. PubMed Central PMCID: PMC2643722. 10.1128/JVI.01785-08 19091863 PMC2643722

[16] Wyrwicz LS, Rychlewski L. Identification of Herpes TATT-binding protein. Antiviral Res. 2007;75(2):167–72. Epub 2007/04/03. 10.1016/j.antiviral.2007.03.002 17400302

[17] Serio TR, Cahill N, Prout ME, Miller G. A functionally distinct TATA box required for late progression through the Epstein-Barr virus life cycle. J Virol. 1998;72(10):8338–43. 9733880 10.1128/jvi.72.10.8338-8343.1998 PMC110205

[18] Davis ZH, Verschueren E, Jang GM, Kleffman K, Johnson JR, Park J, et al Global mapping of herpesvirus-host protein complexes reveals a transcription strategy for late genes. Mol Cell. 2015;57(2):349–60. PubMed Central PMCID: PMCPMC4305015. 10.1016/j.molcel.2014.11.026 25544563 PMC4305015

[19] Davis ZH, Hesser C, Park J, Glaunsinger BA. Interaction Between ORF24 and ORF34 in the Kaposi's Sarcoma-Associated Herpesvirus Late Gene Transcription Factor Complex is Essential For Viral Late Gene Expression. J Virol. 2015. 10.1128/JVI.02157-15 PMC4702566 26468530

[20] Perng YC, Campbell JA, Lenschow DJ, Yu D. Human cytomegalovirus pUL79 is an elongation factor of RNA polymerase II for viral gene transcription. PLoS Pathog. 2014;10(8):e1004350 PubMed Central PMCID: PMC4148446. 10.1371/journal.ppat.1004350 25166009 PMC4148446

[21] Omoto S, Mocarski ES. Transcription of true late (gamma2) cytomegalovirus genes requires UL92 function that is conserved among beta- and gammaherpesviruses. J Virol. 2014;88(1):120–30. PubMed Central PMCID: PMCPMC3911701. 10.1128/JVI.02983-13 24131715 PMC3911701

[22] Sugimoto A, Sato Y, Kanda T, Murata T, Narita Y, Kawashima D, et al Different distributions of Epstein-Barr virus early and late gene transcripts within viral replication compartments. J Virol. 2013;87(12):6693–9. Epub 2013/04/05. PubMed Central PMCID: PMC3676136. 10.1128/JVI.00219-13 23552415 PMC3676136

[23] van Gent M, Braem SG, de Jong A, Delagic N, Peeters JG, Boer IG, et al Epstein-Barr virus large tegument protein BPLF1 contributes to innate immune evasion through interference with toll-like receptor signaling. PLoS Pathog. 2014;10(2):e1003960 PubMed Central PMCID: PMC3930590. 10.1371/journal.ppat.1003960 24586164 PMC3930590

[24] Kumar R, Whitehurst CB, Pagano JS. The Rad6/18 ubiquitin complex interacts with the Epstein-Barr virus deubiquitinating enzyme, BPLF1, and contributes to virus infectivity. J Virol. 2014;88(11):6411–22. PubMed Central PMCID: PMC4093873. 10.1128/JVI.00536-14 24672041 PMC4093873

[25] Saito S, Murata T, Kanda T, Isomura H, Narita Y, Sugimoto A, et al Epstein-Barr virus deubiquitinase downregulates TRAF6-mediated NF-kappaB signaling during productive replication. J Virol. 2013;87(7):4060–70. Epub 2013/02/01. PubMed Central PMCID: PMC3624234. 10.1128/JVI.02020-12 23365429 PMC3624234

[26] Jochum S, Moosmann A, Lang S, Hammerschmidt W, Zeidler R. The EBV immunoevasins vIL-10 and BNLF2a protect newly infected B cells from immune recognition and elimination. PLoS Pathog. 2012;8(5):e1002704 PubMed Central PMCID: PMC3355093. 10.1371/journal.ppat.1002704 22615564 PMC3355093

[27] Zeidler R, Eissner G, Meissner P, Uebel S, Tampe R, Lazis S, et al Downregulation of TAP1 in B lymphocytes by cellular and Epstein-Barr virus-encoded interleukin-10. Blood. 1997;90(6):2390–7. 9310490

[28] Mortazavi A, Williams BA, McCue K, Schaeffer L, Wold B. Mapping and quantifying mammalian transcriptomes by RNA-Seq. Nature methods. 2008;5(7):621–8. Epub 2008/06/03. 10.1038/nmeth.1226 18516045 PMC13303166

[29] Zhang Z, Lopez-Giraldez F, Townsend JP. LOX: inferring Level Of eXpression from diverse methods of census sequencing. Bioinformatics. 2010;26(15):1918–9. Epub 2010/06/12. PubMed Central PMCID: PMC2905554. 10.1093/bioinformatics/btq303 20538728 PMC2905554

[30] Yuan J, Cahir-McFarland E, Zhao B, Kieff E. Virus and cell RNAs expressed during Epstein-Barr virus replication. J Virol. 2006;80(5):2548–65. Epub 2006/02/14. PubMed Central PMCID: PMC1395376. 10.1128/JVI.80.5.2548-2565.2006 16474161 PMC1395376

[31] Summers WC, Klein G. Inhibition of Epstein-Barr virus DNA synthesis and late gene expression by phosphonoacetic acid. J Virol. 1976;18(1):151–5. 176457 10.1128/jvi.18.1.151-155.1976 PMC515533

[32] Tsurumi T, Yamada H, Daikoku T, Yamashita Y, Nishiyama Y. Strand displacement associated DNA synthesis catalyzed by the Epstein-Barr virus DNA polymerase. Biochem Biophys Res Commun. 1997;238(1):33–8. 10.1006/bbrc.1997.7234 9299446

[33] Neuhierl B, Delecluse HJ. The Epstein-Barr virus BMRF1 gene is essential for lytic virus replication. J Virol. 2006;80(10):5078–81. Epub 2006/04/28. PubMed Central PMCID: PMC1472063. 10.1128/JVI.80.10.5078-5081.2006 16641300 PMC1472063

[34] Chen LW, Chang PJ, Delecluse HJ, Miller G. Marked variation in response of consensus binding elements for the Rta protein of Epstein-Barr virus. J Virol. 2005;79(15):9635–50. PubMed Central PMCID: PMC1181578. 10.1128/JVI.79.15.9635-9650.2005 16014926 PMC1181578

[35] Gruffat H, Sergeant A. Characterization of the DNA-binding site repertoire for the Epstein- Barr virus transcription factor R. Nucleic Acids Res. 1994;22(7):1172–8. 8165130 10.1093/nar/22.7.1172 PMC523639

[36] Gruffat H, Manet E, Rigolet A, Sergeant A. The enhancer factor R of Epstein-Barr virus (EBV) is a sequence- specific DNA binding protein. Nucleic Acids Res. 1990;18(23):6835–43. 2175879 10.1093/nar/18.23.6835 PMC332739

[37] Heilmann AM, Calderwood MA, Portal D, Lu Y, Johannsen E. Genome-wide analysis of epstein-barr virus rta DNA binding. J Virol. 2012;86(9):5151–64. Epub 2012/03/02. 10.1128/JVI.06760-11 22379087 PMC3347379

[38] El-Guindy A, Ghiassi-Nejad M, Golden S, Delecluse HJ, Miller G. Essential role of rta in lytic DNA replication of epstein-barr virus. J Virol. 2013;87(1):208–23. PubMed Central PMCID: PMC3536415. 10.1128/JVI.01995-12 23077295 PMC3536415

[39] Arumugaswami V, Wu TT, Martinez-Guzman D, Jia Q, Deng H, Reyes N, et al ORF18 is a transfactor that is essential for late gene transcription of a gammaherpesvirus. J Virol. 2006;80(19):9730–40. Epub 2006/09/16. PubMed Central PMCID: PMC1617240. 10.1128/JVI.00246-06 16973577 PMC1617240

[40] Wong E, Wu TT, Reyes N, Deng H, Sun R. Murine gammaherpesvirus 68 open reading frame 24 is required for late gene expression after DNA replication. J Virol. 2007;81(12):6761–4. Epub 2007/03/30. PubMed Central PMCID: PMC1900117. 10.1128/JVI.02726-06 17392360 PMC1900117

[41] Perng YC, Qian Z, Fehr AR, Xuan B, Yu D. The human cytomegalovirus gene UL79 is required for the accumulation of late viral transcripts. J Virol. 2011;85(10):4841–52. Epub 2011/03/04. PubMed Central PMCID: PMC3126216. 10.1128/JVI.02344-10 21367901 PMC3126216

[42] Omoto S, Mocarski ES. Cytomegalovirus UL91 is essential for transcription of viral true late (gamma2) genes. J Virol. 2013;87(15):8651–64. PubMed Central PMCID: PMCPMC3719799. 10.1128/JVI.01052-13 23720731 PMC3719799

[43] Li R, Zhu J, Xie Z, Liao G, Liu J, Chen MR, et al Conserved Herpesvirus Kinases Target the DNA Damage Response Pathway and TIP60 Histone Acetyltransferase to Promote Virus Replication. Cell host & microbe. 2011;10(4):390–400. Epub 2011/10/25. 22018239 10.1016/j.chom.2011.08.013 PMC3253558

[44] Squatrito M, Gorrini C, Amati B. Tip60 in DNA damage response and growth control: many tricks in one HAT. Trends Cell Biol. 2006;16(9):433–42. Epub 2006/08/15. 10.1016/j.tcb.2006.07.007 16904321

[45] Sun Y, Jiang X, Price BD. Tip60: connecting chromatin to DNA damage signaling. Cell cycle. 2010;9(5):930–6. Epub 2010/02/18. PubMed Central PMCID: PMC2901859. 10.4161/cc.9.5.10931 20160506 PMC2901859

[46] Zacny VL, Wilson J, Pagano JS. The Epstein-Barr virus immediate-early gene product, BRLF1, interacts with the retinoblastoma protein during the viral lytic cycle. J Virol. 1998;72(10):8043–51. 9733844 10.1128/jvi.72.10.8043-8051.1998 PMC110141

[47] Countryman J, Gradoville L, Bhaduri-McIntosh S, Ye J, Heston L, Himmelfarb S, et al Stimulus duration and response time independently influence the kinetics of lytic cycle reactivation of Epstein-Barr virus. J Virol. 2009;83(20):10694–709. Epub 2009/08/07. PubMed Central PMCID: PMC2753116. 10.1128/JVI.01172-09 19656890 PMC2753116

[48] Wang JT, Yang PW, Lee CP, Han CH, Tsai CH, Chen MR. Detection of Epstein-Barr virus BGLF4 protein kinase in virus replication compartments and virus particles. J Gen Virol. 2005;86(Pt 12):3215–25. Epub 2005/11/22. 10.1099/vir.0.81313-0 16298966

[49] Daikoku T, Kudoh A, Fujita M, Sugaya Y, Isomura H, Shirata N, et al Architecture of replication compartments formed during Epstein-Barr virus lytic replication. J Virol. 2005;79(6):3409–18. 10.1128/JVI.79.6.3409-3418.2005 15731235 PMC1075702

[50] Park R, Heston L, Shedd D, Delecluse HJ, Miller G. Mutations of amino acids in the DNA-recognition domain of Epstein-Barr virus ZEBRA protein alter its sub-nuclear localization and affect formation of replication compartments. Virology. 2008;382(2):145–62. Epub 2008/10/22. PubMed Central PMCID: PMC2654287. 10.1016/j.virol.2008.09.009 18937960 PMC2654287

[51] Ragoczy T, Miller G. Role of the epstein-barr virus RTA protein in activation of distinct classes of viral lytic cycle genes. J Virol. 1999;73(12):9858–66. 10559298 10.1128/jvi.73.12.9858-9866.1999 PMC113035

[52] El-Guindy AS, Miller G. Phosphorylation of Epstein-Barr virus ZEBRA protein at its casein kinase 2 sites mediates its ability to repress activation of a viral lytic cycle late gene by Rta. J Virol. 2004;78(14):7634–44. 10.1128/JVI.78.14.7634-7644.2004 15220438 PMC434091

[53] Yang YC, Chang LK. Role of TAF4 in transcriptional activation by Rta of Epstein-Barr Virus. PLoS One. 2013;8(1):e54075 PubMed Central PMCID: PMCPMC3542328. 10.1371/journal.pone.0054075 23326574 PMC3542328

[54] Manet E, Allera C, Gruffat H, Mikaelian I, Rigolet A, Sergeant A. The acidic activation domain of the Epstein-Barr virus transcription factor R interacts in vitro with both TBP and TFIIB and is cell- specifically potentiated by a proline-rich region. Gene Expr. 1993;3(1):49–59. 8389627 PMC6081622

[55] Ragoczy T, Miller G. Autostimulation of the Epstein-Barr virus BRLF1 promoter is mediated through consensus Sp1 and Sp3 binding sites. J Virol. 2001;75(11):5240–51. 10.1128/JVI.75.11.5240-5251.2001 11333906 PMC114930

[56] Chang LK, Chung JY, Hong YR, Ichimura T, Nakao M, Liu ST. Activation of Sp1-mediated transcription by Rta of Epstein-Barr virus via an interaction with MCAF1. Nucleic Acids Res. 2005;33(20):6528–39. PubMed Central PMCID: PMC1298921. 10.1093/nar/gki956 16314315 PMC1298921

[57] Chua HH, Lee HH, Chang SS, Lu CC, Yeh TH, Hsu TY, et al Role of the TSG101 gene in Epstein-Barr virus late gene transcription. J Virol. 2007;81(5):2459–71. Epub 2006/12/22. PubMed Central PMCID: PMC1865947. 10.1128/JVI.02289-06 17182691 PMC1865947

[58] Adamson AL, Darr D, Holley-Guthrie E, Johnson RA, Mauser A, Swenson J, et al Epstein-Barr virus immediate-early proteins BZLF1 and BRLF1 activate the ATF2 transcription factor by increasing the levels of phosphorylated p38 and c-Jun N-terminal kinases. J Virol. 2000;74(3):1224–33. PubMed Central PMCID: PMC111456. 10627532 10.1128/jvi.74.3.1224-1233.2000 PMC111456

[59] Ressing ME, van Gent M, Gram AM, Hooykaas MJ, Piersma SJ, Wiertz EJ. Immune Evasion by Epstein-Barr Virus. Curr Top Microbiol Immunol. 2015;391:355–81. 10.1007/978-3-319-22834-1_12 26428381

[60] Schmaus S, Wolf H, Schwarzmann F. The reading frame BPLF1 of Epstein-Barr virus: a homologue of herpes simplex virus protein VP16. Virus Genes. 2004;29(2):267–77. Epub 2004/07/31. 10.1023/B:VIRU.0000036387.39937.9b 15284487

[61] Hudson GS, Bankier AT, Satchwell SC, Barrell BG. The short unique region of the B95-8 Epstein-Barr virus genome. Virology. 1985;147(1):81–98. 2998073 10.1016/0042-6822(85)90229-6

[62] Jochum S, Ruiss R, Moosmann A, Hammerschmidt W, Zeidler R. RNAs in Epstein-Barr virions control early steps of infection. Proc Natl Acad Sci U S A. 2012;109(21):E1396–404. Epub 2012/05/01. PubMed Central PMCID: PMC3361417. 10.1073/pnas.1115906109 22543160 PMC3361417

[63] Miyazaki I, Cheung RK, Dosch HM. Viral interleukin 10 is critical for the induction of B cell growth transformation by Epstein-Barr virus. The Journal of experimental medicine. 1993;178(2):439–47. Epub 1993/08/01. PubMed Central PMCID: PMC2191114. 8393476 10.1084/jem.178.2.439 PMC2191114

[64] Bejarano MT, Masucci MG. Interleukin-10 abrogates the inhibition of Epstein-Barr virus-induced B-cell transformation by memory T-cell responses. Blood. 1998;92(11):4256–62. 9834231

[65] Whitehurst CB, Ning S, Bentz GL, Dufour F, Gershburg E, Shackelford J, et al The Epstein-Barr virus (EBV) deubiquitinating enzyme BPLF1 reduces EBV ribonucleotide reductase activity. J Virol. 2009;83(9):4345–53. Epub 2009/02/27. PubMed Central PMCID: PMC2668490. 10.1128/JVI.02195-08 19244336 PMC2668490

[66] Gredmark-Russ S, Isaacson MK, Kattenhorn L, Cheung EJ, Watson N, Ploegh HL. A gammaherpesvirus ubiquitin-specific protease is involved in the establishment of murine gammaherpesvirus 68 infection. J Virol. 2009;83(20):10644–52. PubMed Central PMCID: PMCPMC2753118. 10.1128/JVI.01017-09 19706716 PMC2753118

[67] Gastaldello S, Hildebrand S, Faridani O, Callegari S, Palmkvist M, Di Guglielmo C, et al A deneddylase encoded by Epstein-Barr virus promotes viral DNA replication by regulating the activity of cullin-RING ligases. Nat Cell Biol. 2010;12(4):351–61. 10.1038/ncb2035 20190741

[68] Francis AL, Gradoville L, Miller G. Alteration of a single serine in the basic domain of the Epstein-Barr virus ZEBRA protein separates its functions of transcriptional activation and disruption of latency. J Virol. 1997;71:3054–61. 9060666 10.1128/jvi.71.4.3054-3061.1997 PMC191435

[69] Ragoczy T, Heston L, Miller G. The Epstein-Barr virus Rta protein activates lytic cycle genes and can disrupt latency in B lymphocytes. J Virol. 1998;72(10):7978–84. 9733836 10.1128/jvi.72.10.7978-7984.1998 PMC110133

[70] Chen MR, Chang SJ, Huang H, Chen JY. A protein kinase activity associated with Epstein-Barr virus BGLF4 phosphorylates the viral early antigen EA-D in vitro. J Virol. 2000;74(7):3093–104. Epub 2000/03/09. PubMed Central PMCID: PMC111808. 10708424 10.1128/jvi.74.7.3093-3104.2000 PMC111808

[71] Feederle R, Kost M, Baumann M, Janz A, Drouet E, Hammerschmidt W, et al The Epstein-Barr virus lytic program is controlled by the co-operative functions of two transactivators. EMBO J. 2000;19(12):3080–9. 10.1093/emboj/19.12.3080 10856251 PMC203345

[72] Delecluse HJ, Hilsendegen T, Pich D, Zeidler R, Hammerschmidt W. Propagation and recovery of intact, infectious Epstein-Barr virus from prokaryotic to human cells. Proc Natl Acad Sci U S A. 1998;95(14):8245–50. 9653172 10.1073/pnas.95.14.8245 PMC20961

[73] Heston L, Rabson M, Brown N, Miller G. New Epstein-Barr virus variants from cellular subclones of P3J-HR-1 Burkitt lymphoma. Nature. 1982;295(5845):160–3. 6276755 10.1038/295160a0

[74] Pearson GR, Vroman B, Chase B, Sculley T, Hummel M, Kieff E. Identification of polypeptide components of the Epstein-Barr virus early antigen complex with monoclonal antibodies. J Virol. 1983;47(1):193–201. 6306272 10.1128/jvi.47.1.193-201.1983 PMC255226

[75] Trapnell C, Pachter L, Salzberg SL. TopHat: discovering splice junctions with RNA-Seq. Bioinformatics. 2009;25(9):1105–11. Epub 2009/03/18. PubMed Central PMCID: PMC2672628. 10.1093/bioinformatics/btp120 19289445 PMC2672628

[76] O'Grady T, Cao S, Strong MJ, Concha M, Wang X, Splinter Bondurant S, et al Global Bidirectional Transcription of the Epstein-Barr Virus Genome during Reactivation. J Virol. 2014;88(3):1604–16. Epub 2013/11/22. PubMed Central PMCID: PMC3911580. 10.1128/JVI.02989-13 24257595 PMC3911580

[77] Li B, Dewey CN. RSEM: accurate transcript quantification from RNA-Seq data with or without a reference genome. BMC Bioinformatics. 2011;12:323 Epub 2011/08/06. PubMed Central PMCID: PMC3163565. 10.1186/1471-2105-12-323 21816040 PMC3163565

[78] Langmead B, Salzberg SL. Fast gapped-read alignment with Bowtie 2. Nature methods. 2012;9(4):357–9. Epub 2012/03/06. PubMed Central PMCID: PMC3322381. 10.1038/nmeth.1923 22388286 PMC3322381

[79] Leng N, Dawson JA, Thomson JA, Ruotti V, Rissman AI, Smits BM, et al EBSeq: an empirical Bayes hierarchical model for inference in RNA-seq experiments. Bioinformatics. 2013;29(8):1035–43. Epub 2013/02/23. PubMed Central PMCID: PMC3624807. 10.1093/bioinformatics/btt087 23428641 PMC3624807

[80] Li H, Durbin R. Fast and accurate long-read alignment with Burrows-Wheeler transform. Bioinformatics. 2010;26(5):589–95. PubMed Central PMCID: PMCPMC2828108. 10.1093/bioinformatics/btp698 20080505 PMC2828108

[81] Robinson JT, Thorvaldsdottir H, Winckler W, Guttman M, Lander ES, Getz G, et al Integrative genomics viewer. Nat Biotechnol. 2011;29(1):24–6. PubMed Central PMCID: PMCPMC3346182. 10.1038/nbt.1754 21221095 PMC3346182

[82] Thorvaldsdottir H, Robinson JT, Mesirov JP. Integrative Genomics Viewer (IGV): high-performance genomics data visualization and exploration. Brief Bioinform. 2013;14(2):178–92. PubMed Central PMCID: PMCPMC3603213. 10.1093/bib/bbs017 22517427 PMC3603213

