# Supplementary figures and images for: The Epstein-Barr Virus Immunoevasins BCRF1 and BPLF1 Are Expressed by a Mechanism Independent of the Canonical Late Pre-initiation Complex
Source: PLoS Pathog. 2016 Nov 17;12(11):e1006008. doi: 10.1371/journal.ppat.1006008 (PMC5113994; doi:10.1371/journal.ppat.1006008)

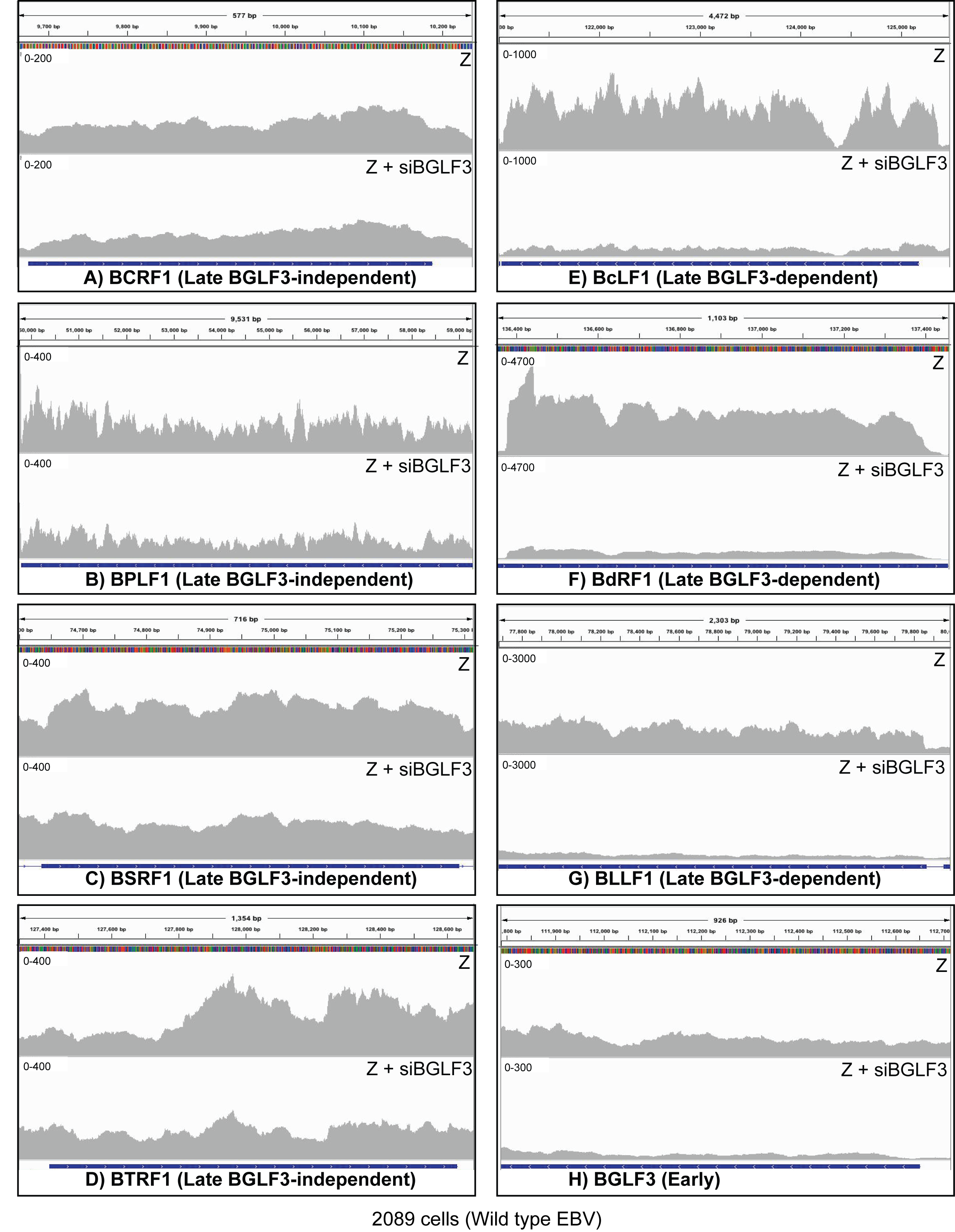

Supplement: S1 Fig — Graphs generated by the Integrative Genomics Viewer (IGV) visualization tool [81, 82] compare the effect of siBGLF3 on the number of reads mapping to the four BGLF3-independent late transcripts (A to D), three BGLF3-dependent late transcripts (E to G) and the BGLF3 transcript (H). The Bam files used to plot these graphs were obtained from RNA-seq data assessing the effect of siBGLF3 on viral gene expression during the lytic cycle (Fig 1). The data set range is indicated at the top left corner of each graph. (TIF) [file ppat.1006008.s001.tif]

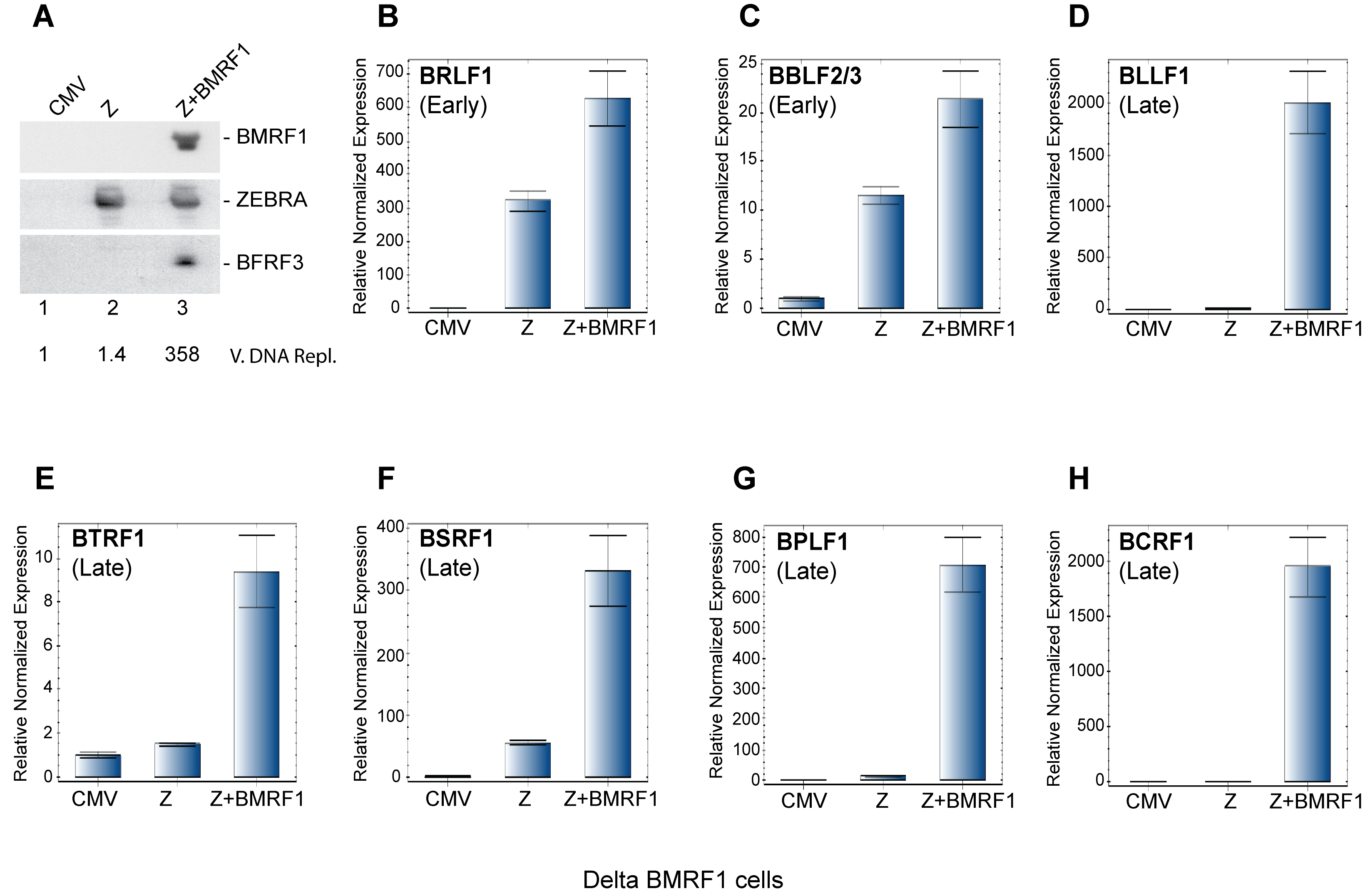

Supplement: S2 Fig — A) Western blot analysis assessing the expression of BMRF1 (early), ZEBRA and BFRF3 (late) in delta BMRF1 cells transfected with empty vector (CMV), ZEBRA (Z), or ZEBRA (Z) plus BMRF1. The extent of intracellular viral DNA replication (V. DNA Repl.) was assessed in the absence and presence of BMRF1. Total DNA was prepared from the same samples and analyzed by qPCR using primers specific to EBV oriLyt. B-H) RT-qPCR measuring the level of seven lytic transcripts: BRLF1 (early), BBLF2/3 (early), BLLF1 (BGLF3-dependent late), BTRF1 (BGLF3-independent late), BSRF1 (BGLF3-independent late), BPLF1 (BGLF3-independent late), and BCRF1 (BGLF3-independent late). Lack of BMRF1, an essential replication protein, abolished expression of the four BGLF3-independent genes. (TIF) [file ppat.1006008.s002.tif]

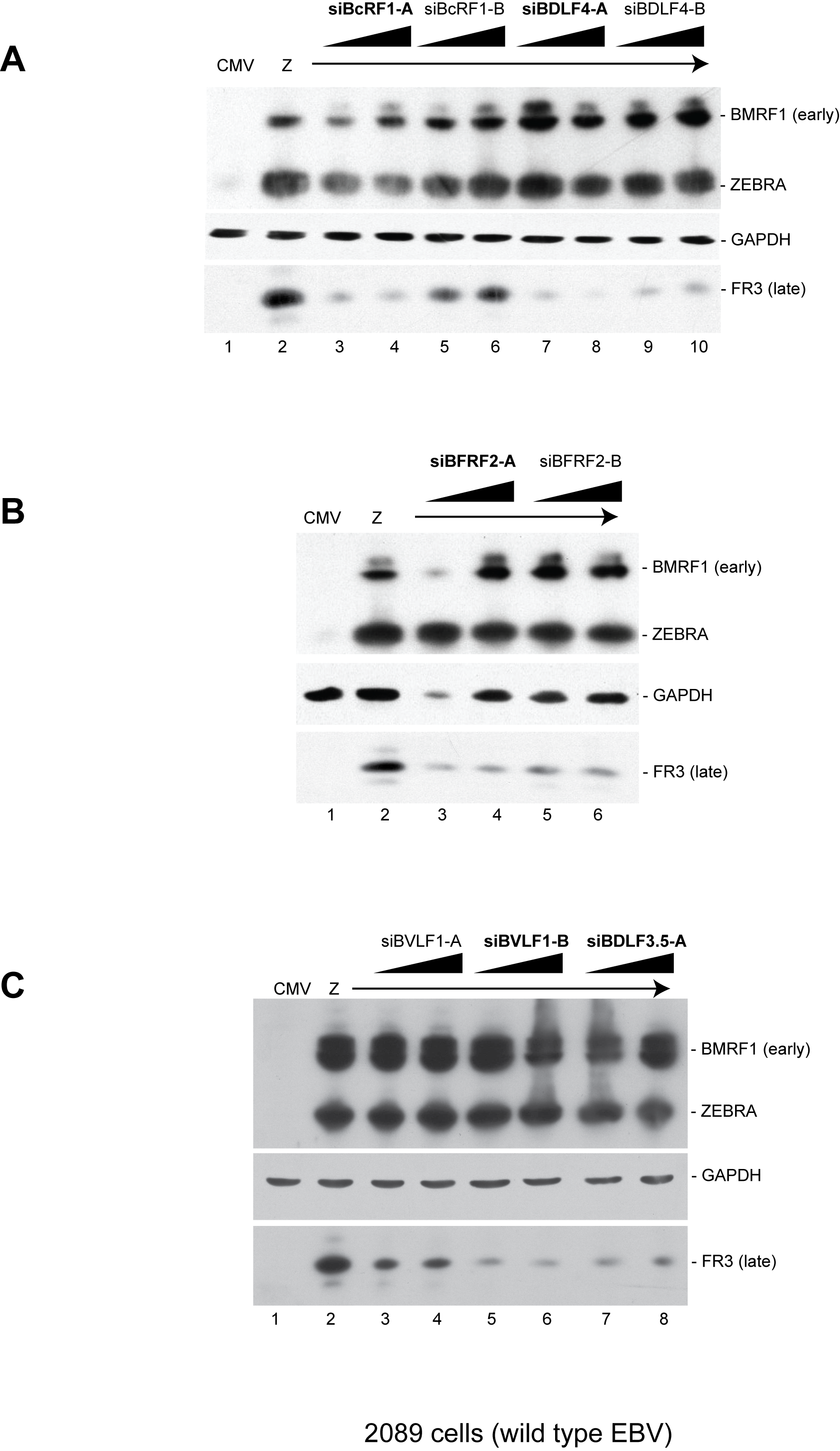

Supplement: S3 Fig — Western blot analysis assessing the effect of siRNAs to BcRF1, BDLF4 (panel A), BFRF2 (panel B), BVLF1, and BDLF3.5 (panel C) on expression of the late FR3 protein. The effect of each siRNA was studied at two concentrations, 40 nM and 80 nM. siRNAs marked in bold font were employed in subsequent analyses. (TIF) [file ppat.1006008.s003.tif]

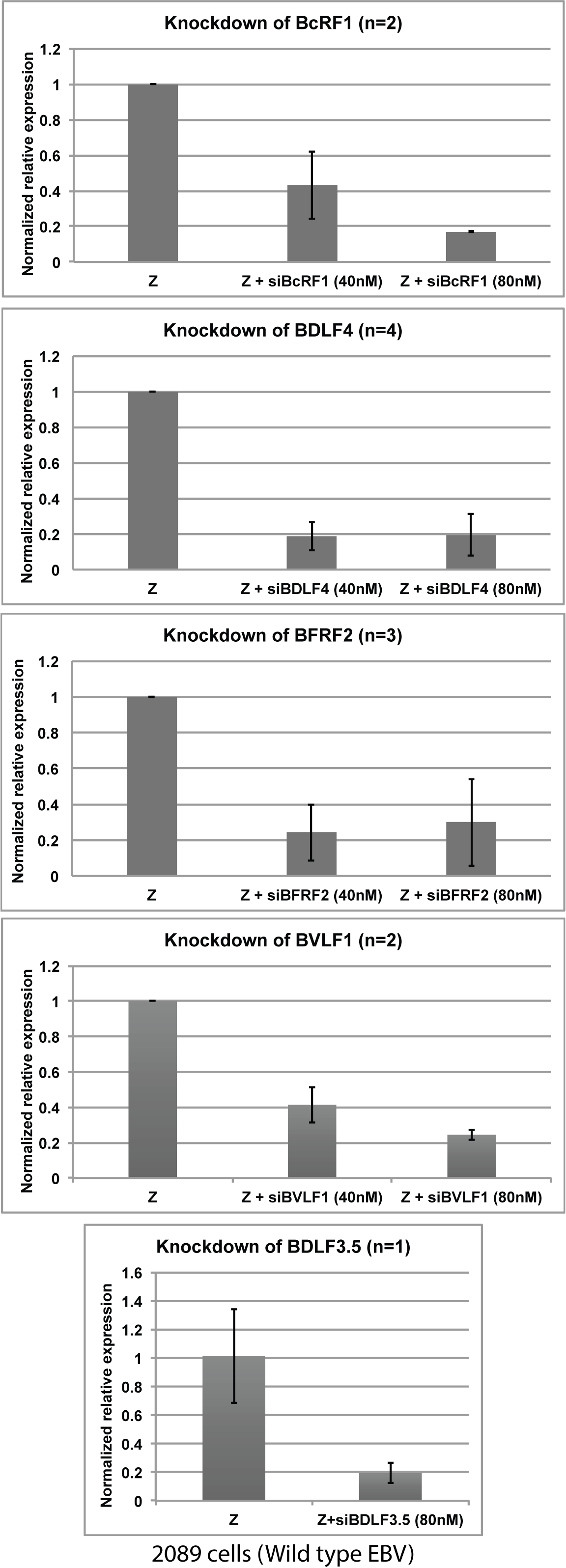

Supplement: S4 Fig — 2089 cells were transfected with empty vector (CMV), ZEBRA (Z) alone, or ZEBRA plus the indicated siRNA. Cells were harvested after 48h and total RNA was purified. The abundance of five lytic transcripts encoding BcRF1, BVLF1, BDLF4, BFRF2, and BDLF3.5 was quantitated using RT-qPCR. (TIF) [file ppat.1006008.s004.tif]

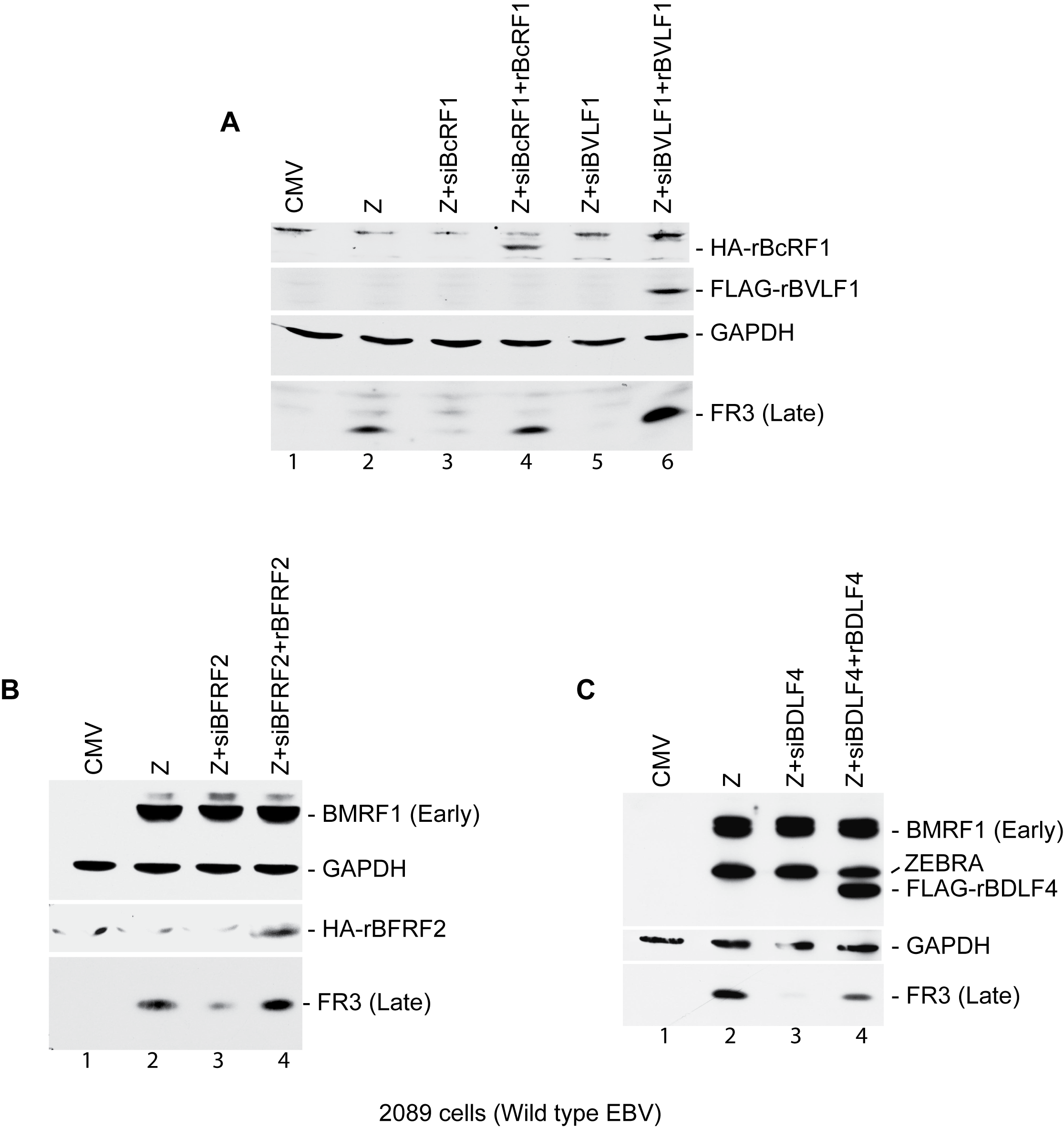

Supplement: S5 Fig — To assess the specificity of each of the siRNAs generated towards the components of vPIC, we introduced silent mutations that disrupt the ability of each siRNA to bind to its corresponding mRNA. The following four siRNA-resistant forms were generated: rBcRF1, rBVLF1, rBFRF2, and rBDLF4. The experiment was performed in 2089 cells carrying wild type EBV. Panel A shows the capacity of rBcRF1 and rBVLF1 to restore expression of the late FR3 capsid protein in the presence of siBcRF1 and siBVLF1, respectively. Panels B and C demonstrate the capacity of rBFRF2 and rBDLF4 to rescue expression of late genes in the presence of their corresponding siRNA. rBcRF1 and rBFRF2 are HA-tagged and were detected using HA antibody. rBVLF1 and rBDLF4 are FLAG-tagged and were detected using a FLAG antibody. (TIF) [file ppat.1006008.s005.tif]

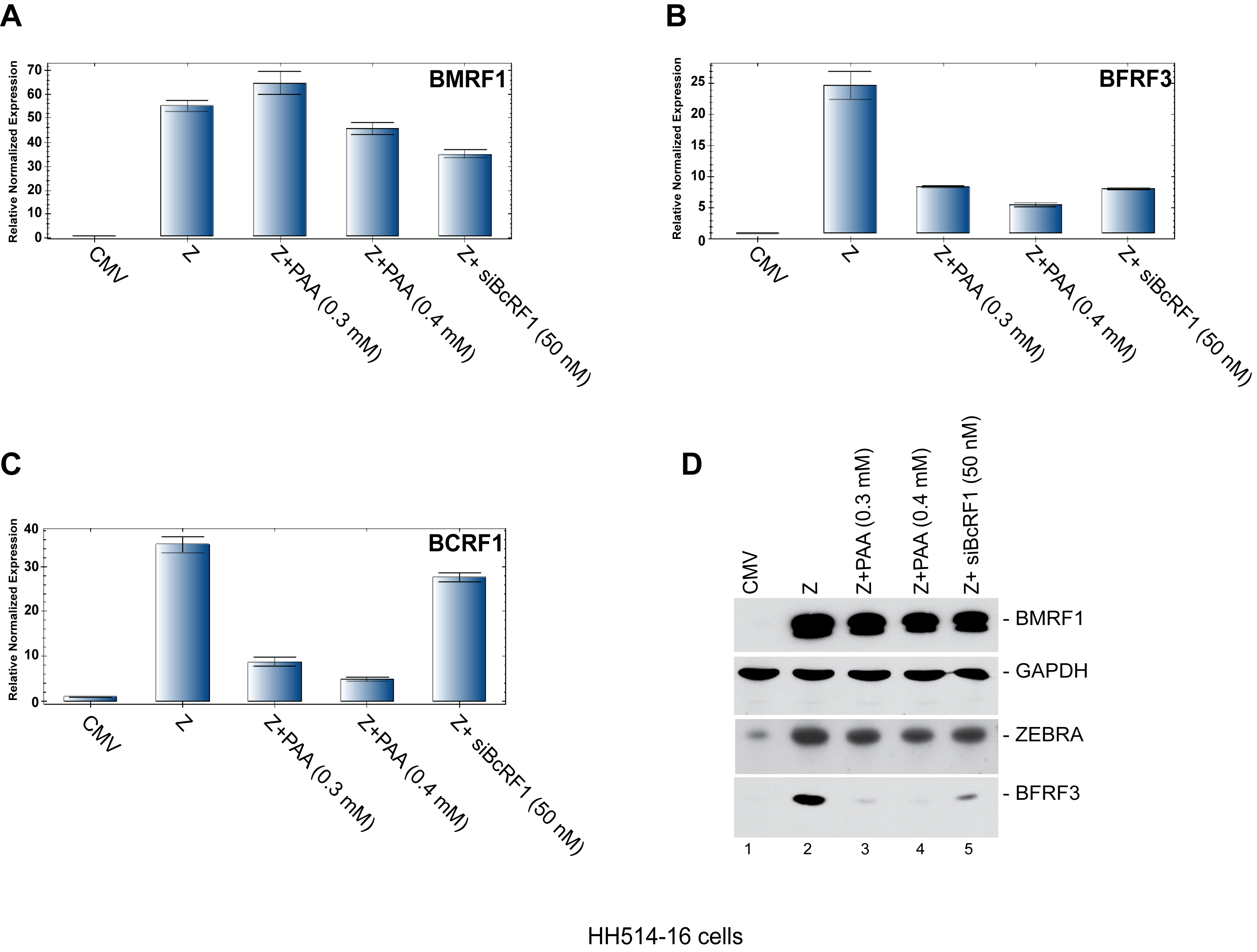

Supplement: S6 Fig — A-C) RT-qPCR assessing the kinetics of BCRF1 (vIL10) and its dependence on vTBP in HH514-16 Burkitt lymphoma cells. HH514-16 cells were transfected with empty vector (CMV) or ZEBRA (Z) to induce the lytic cycle. ZEBRA-transfected cells were treated with two concentrations (0.3 and 0.4 mM) of phosphonoacetic acid (PAA) or co-transfected with siBcRF1 (50 nM). Panel D shows a Western blot of cell lysates prepared from the same samples analyzed in panels A, B and C. The membrane was immunoblotted with antibodies to BMRF1, GAPDH, ZEBRA (Z), and BFRF3 (FR3). (TIF) [file ppat.1006008.s006.tif]

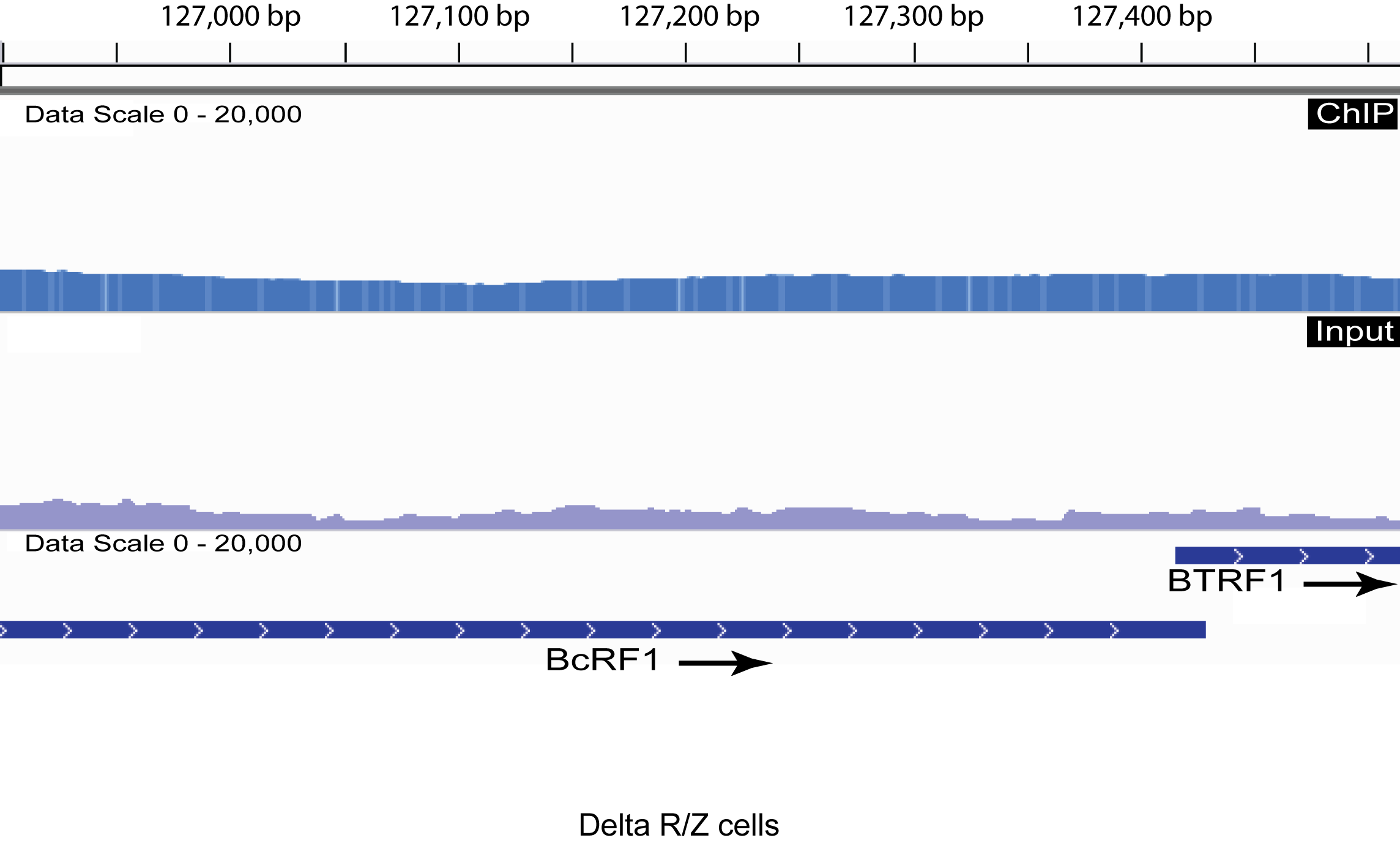

Supplement: S7 Fig — Graphs generated by the Integrative Genomics Viewer (IGV) visualization tool [81, 82] shows Rta ChIP (dark blue) and Input (light blue) tracks of the region upstream of BTRF1 gene. (TIF) [file ppat.1006008.s007.tif]
