# Supplementary material for: The Epstein-Barr Virus Immunoevasins BCRF1 and BPLF1 Are Expressed by a Mechanism Independent of the Canonical Late Pre-initiation Complex
Source: PLoS Pathog. 2016 Nov 17;12(11):e1006008. doi: 10.1371/journal.ppat.1006008 (PMC5113994; doi:10.1371/journal.ppat.1006008)
Supplement: S1 Table — (DOCX) [file ppat.1006008.s008.docx]

**S1 Table: Analysis of RT-qPCR data studying the effect of siRNAs to late gene regulators on expression of select EBV genes.**

**siBGLF3**

| **Gene**  **(*G*)** | **Average Fold Stimulation (Z/CMV)^a^** | | **Change in Expression (Z+siRNA/Z)^b^** | | **Z versus Z+siRNA**  **(*p*-value)^c^** | | ***G* versus *G3D*-Late Genes**  **(*p-*value)^d^** | |
| --- | --- | --- | --- | --- | --- | --- | --- | --- |
| BMRF1 | 1565.62 | 1.35 | | 0.1270 | | 0.0001 | |  |
| BRLF1 | 1249.72 | 1.90 | | 0.0296 | | <0.0001 | |  |
| BdRF1 | 3463.94 | 0.19 | | 0.0068 | | 0.903 | |  |
| BLLF1 | 5177.33 | 0.31 | | 0.0192 | | 0.737 | |  |
| BFRF3 | 979.16 | 0.25 | | 0.0124 | | 0.9268 | |  |
| BLRF2 | 7886.29 | 0.13 | | 0.0018 | | 0.7572 | |  |
| BTRF1 | 20.78 | 1.86 | | 0.0049 | | <0.0001 | |  |
| BPLF1 | 744.88 | 0.97 | | 0.7000 | | 0.0102 | |  |
| BCRF1 | 7431.51 | 1.39 | | 0.4400 | | 0.0004 | |  |
| BSRF1 | 421.65 | 0.93 | | 0.0998 | | 0.0427 | |  |

**siBcRF1**

| **Gene**  **(*G*)** | **Average Fold Stimulation (Z/CMV)^a^** | | **Change in Expression (Z+siRNA/Z)^b^** | | **Z versus Z+siRNA**  **(*p*-value)^c^** | | ***G* versus *G3D*-Late Genes**  **(*p-*value)^d^** | |
| --- | --- | --- | --- | --- | --- | --- | --- | --- |
| BMRF1 | 641.78 | 0.76 | | 0.2949 | | 0.0012 | |  |
| BRLF1 | 94.99 | 1.25 | | 0.3716 | | <0.0001 | |  |
| BdRF1 | 1211.36 | 0.09 | | 0.0022 | | 0.6835 | |  |
| BLLF1 | 490.22 | 0.20 | | 0.0792 | | 0.8327 | |  |
| BFRF3 | 1115.86 | 0.17 | | 0.0083 | | 0.9386 | |  |
| BLRF2 | 2293.99 | 0.18 | | 0.0222 | | 0.8987 | |  |
| BTRF1 | 63.75 | 0.43 | | 0.0013 | | 0.118 | |  |
| BPLF1 | 536.77 | 0.86 | | 0.5355 | | 0.0003 | |  |
| BCRF1 | 1522.21 | 0.63 | | 0.1153 | | 0.0044 | |  |
| BSRF1 | 715.42 | 0.58 | | 0.2119 | | 0.0167 | |  |

**siBDLF4**

| **Gene**  **(*G*)** | **Average Fold Stimulation (Z/CMV)^a^** | | **Change in Expression (Z+siRNA/Z)^b^** | | **Z versus Z+siRNA**  **(*p*-value)^c^** | | ***G* versus *G3D*-Late Genes**  **(*p-*value)^d^** | |
| --- | --- | --- | --- | --- | --- | --- | --- | --- |
| BMRF1 | 624.69 | 0.89 | | 0.3764 | | 0.0015 | |  |
| BRLF1 | 109.48 | 1.50 | | 0.4216 | | <0.0001 | |  |
| BdRF1 | 1767.47 | 0.11 | | 0.0004 | | 0.8801 | |  |
| BLLF1 | 1666.37 | 0.12 | | 0.0016 | | 0.9185 | |  |
| BFRF3 | 1182.46 | 0.19 | | 0.0005 | | 0.823 | |  |
| BLRF2 | 2004.59 | 0.16 | | 0.001 | | 0.9451 | |  |
| BTRF1 | 93.06 | 1.32 | | 0.3769 | | <0.0001 | |  |
| BPLF1 | 592.06 | 0.71 | | 0.054 | | 0.0191 | |  |
| BCRF1 | 1899.48 | 0.78 | | 0.1388 | | 0.0042 | |  |
| BSRF1 | 677.71 | 0.65 | | 0.0806 | | 0.0274 | |  |

**siBFRF2**

| **Gene**  **(*G*)** | **Average Fold Stimulation (Z/CMV)^a^** | | **Change in Expression (Z+siRNA/Z)^b^** | | **Z versus Z+siRNA**  **(*p*-value)^c^** | | ***G* versus *G3D*-Late Genes**  **(*p-*value)^d^** | |
| --- | --- | --- | --- | --- | --- | --- | --- | --- |
| BMRF1 | 558.06 | 0.85 | | 0.3035 | | 0.0002 | |  |
| BRLF1 | 109.50 | 1.32 | | 0.124 | | <0.0001 | |  |
| BdRF1 | 1152.63 | 0.14 | | 0.0042 | | 0.9976 | |  |
| BLLF1 | 1663.51 | 0.18 | | 0.0059 | | 0.8439 | |  |
| BFRF3 | 1343.33 | 0.09 | | 0.0036 | | 0.7804 | |  |
| BLRF2 | 2447.15 | 0.16 | | 0.0087 | | 0.939 | |  |
| BTRF1 | 80.08 | 1.36 | | 0.0456 | | <0.0001 | |  |
| BPLF1 | 512.67 | 0.71 | | 0.0245 | | 0.0012 | |  |
| BCRF1 | 1751.30 | 0.78 | | 0.2122 | | 0.0003 | |  |
| BSRF1 | 499.71 | 0.71 | | 0.1916 | | 0.0011 | |  |

**siBDLF3.5**

| **Gene**  **(*G*)** | **Average Fold Stimulation (Z/CMV)^a^** | | **Change in Expression (Z+siRNA/Z)^b^** | | **Z versus Z+siRNA**  **(*p*-value)^c^** | | ***G* versus *G3D*-Late Genes**  **(*p-*value)^d^** | |
| --- | --- | --- | --- | --- | --- | --- | --- | --- |
| BMRF1 | 5609.00 | 0.86 | | 0.4842 | | 0.0008 | |  |
| BRLF1 | 204.41 | 0.96 | | 0.7308 | | 0.0002 | |  |
| BdRF1 | 1586.77 | 0.14 | | 0.0361 | | 0.889 | |  |
| BLLF1 | 1320.28 | 0.16 | | 0.0119 | | 0.9692 | |  |
| BFRF3 | 1174.22 | 0.21 | | 0.0243 | | 0.8143 | |  |
| BLRF2 | 2329.30 | 0.16 | | 0.0233 | | 0.9547 | |  |
| BTRF1 | 61.35 | 1.21 | | 0.3823 | | <0.0001 | |  |
| BPLF1 | 664.25 | 0.58 | | 0.0144 | | 0.0225 | |  |
| BCRF1 | 1973.97 | 0.95 | | 0.435 | | <0.0001 | |  |
| BSRF1 | 318.45 | 1.01 | | 0.7813 | | <0.0001 | |  |

**siBVLF1**

| **Gene**  **(*G*)** | **Average Fold Stimulation (Z/CMV)^a^** | | **Change in Expression (Z+siRNA/Z)^b^** | | **Z versus Z+siRNA**  **(*p*-value)^c^** | | ***G* versus *G3D*-Late Genes**  **(*p-*value)^d^** | |
| --- | --- | --- | --- | --- | --- | --- | --- | --- |
| BMRF1 | 5609.00 | 0.89 | | 0.5668 | | 0.0055 | |  |
| BRLF1 | 204.44 | 1.27 | | 0.162 | | 0.0001 | |  |
| BdRF1 | 1586.77 | 0.09 | | 0.0114 | | 0.9554 | |  |
| BLLF1 | 1330.46 | 0.09 | | 0.0004 | | 0.9361 | |  |
| BFRF3 | 1174.22 | 0.15 | | 0.0077 | | 0.8614 | |  |
| BLRF2 | 2297.10 | 0.11 | | 0.0008 | | 0.9894 | |  |
| BTRF1 | 61.10 | 1.48 | | 0.3736 | | <0.0001 | |  |
| BPLF1 | 664.27 | 0.60 | | 0.1183 | | 0.0499 | |  |
| BCRF1 | 1973.95 | 1.06 | | 0.8407 | | 0.0005 | |  |
| BSRF1 | 311.70 | 0.76 | | 0.1701 | | 0.0121 | |  |

**siBGLF4**

| **Gene**  **(*G*)** | **Average Fold Stimulation (Z/CMV)^a^** | | **Change in Expression (Z+siRNA/Z)^b^** | | **Z versus Z+siRNA**  **(*p*-value)^c^** | | ***G* versus *G3D*-Late Genes**  **(*p-*value)^d^** | |
| --- | --- | --- | --- | --- | --- | --- | --- | --- |
| BMRF1 | 624.28 | 0.90 | | 0.3824 | | <0.0001 | |  |
| BRLF1 | 331.58 | 0.64 | | 0.0183 | | <0.0001 | |  |
| BdRF1 | 159.30 | 0.13 | | 0.0061 | | 0.9816 | |  |
| BLLF1 | 258.75 | 0.12 | | 0.0175 | | 0.936 | |  |
| BFRF3 | 224.91 | 0.16 | | 0.0065 | | 0.5592 | |  |
| BLRF2 | 982.12 | 0.10 | | 0.0055 | | 0.6324 | |  |
| BTRF1 | 8.87 | 0.34 | | 0.0102 | | 0.002 | |  |
| BPLF1 | 407.24 | 0.21 | | 0.0069 | | 0.1714 | |  |
| BCRF1 | 682.14 | 0.11 | | 0.0046 | | 0.735 | |  |
| BSRF1 | 79.41 | 0.28 | | 0.0609 | | 0.02 | |  |

1. Average fold stimulation was calculated by dividing Z fold-induction by CMV for each data point.
2. Change in Expression (Z+siRNA/Z) assesses the effect of individual siRNAs on the expression of each of the studied genes (***G***).
3. Significant changes in expression of genes (***G***) in cells transfected with Z versus Z+siRNA (t-test*, p-*value ≤ 0.05).
4. Statistical assessment for changes in expression of individual genes (***G***) relative to the average change in expression of four BGLF3-dependent late genes (***G3D-Late Genes*** – BdRF1, BLLF1, BFRF3, and BLRF2) following treatment with siRNA (*ANOVA, p-*value ≤ 0.05).
